# Land use modification causes slow, but predictable, change in soil microbial community composition and functional potential

**DOI:** 10.1186/s40793-023-00485-x

**Published:** 2023-04-06

**Authors:** Z. Louisson, S. M. Hermans, H. L. Buckley, B. S. Case, M. Taylor, F. Curran-Cournane, G. Lear

**Affiliations:** 1 School of Biological Sciences, University of Auckland, 3a Symonds Street, Auckland, 1010 New Zealand; 2 School of Science, Auckland University of Technology, 34 St Paul Street, Auckland, 1010 New Zealand; 3 Waikato Regional Council, 160 Ward St, Hamilton, 3204 New Zealand; 4 Joint Evidence Data and Insights, Ministry for the Environment, 45 Queens Street, Auckland, 1010 New Zealand

**Keywords:** Temporal change, Soil bacteria, Metagenomics, Land management, Soil health, Land use change, 16S rRNA gene

## Abstract

**Background:**

Bacterial communities are critical to ecosystem functioning and sensitive to their surrounding physiochemical environment. However, the impact of land use change on microbial communities remains understudied. We used 16S rRNA gene amplicon sequencing and shotgun metagenomics to assess soil microbial communities' taxonomic and functional responses to land use change. We compared data from long-term grassland, exotic forest and horticulture reference sites to data from sites that transitioned from (i) Grassland to exotic forest or horticulture and from (ii) Exotic forest to grassland.

**Results:**

Community taxonomic and functional profiles of the transitional sites significantly differed from those within reference sites representing both their historic and current land uses (*P* < 0.001). The bacterial communities in sites that transitioned more recently were compositionally more similar to those representing their historic land uses. In contrast, the composition of communities from sites exposed to older conversion events had shifted towards the compositions at reference sites representing their current land use.

**Conclusions:**

Our study indicates that microbial communities respond in a somewhat predictable way after a land use conversion event by shifting from communities reflecting their former land use towards those reflecting their current land use. Our findings help us to better understand the legacy effects of land use change on soil microbial communities and implications for their role in soil health and ecosystem functioning. Understanding the responsiveness of microbial communities to environmental disturbances will aid us in incorporating biotic variables into soil health monitoring techniques in the future.

**Supplementary Information:**

The online version contains supplementary material available at 10.1186/s40793-023-00485-x.

## Background

Land use conversion leaves enduring legacy effects on soil ecosystems and is considered a dominant threat to biodiversity [56, 57]. The impacts on abiotic conditions are well-researched. Soil pH levels, carbon and nitrogen concentrations are highly related to agricultural practices, but it may take decades to centuries for these to reflect their modern-day land use, following conversion [15, 41, 57]. Compared to physicochemical analyses, far less focus has been directed towards the impact of land use change on microbial community composition and functioning. It is important to investigate this further as microbial communities are an essential biological component of all soil ecosystems. Further, they are responsible for fundamental processes, including nutrient cycling, decomposition and soil formation [34, 53]. Soil physicochemical variables, which may be highly impacted by land use [14, 30], are increasingly considered to be dominant determinants of microbial community composition, with different land uses and management types often having distinct microbial community profiles [24, 65].

Land use intensification is often associated with changes in microbial biodiversity [59]; however, the impacts of such changes on microbial community functioning remain understudied [4]. Some studies indicate that reductions in microbial biodiversity do translate to reductions in ecosystem functioning [58] and that biodiversity is important for functional stability [55], while others have observed no significant effects from reduced biodiversity [61, 62]. These discrepancies could be due to the high levels of functional redundancy observed in soil microbial communities where taxa from different clades are responsible for the same or similar functional processes [36], potentially mitigating the effects of biodiversity loss. However, this phenomenon is difficult to confirm in microbial communities using compositional data, largely due to a lack of knowledge surrounding the functional roles of different microbial taxa [1]. Consequently, when analysing the impact of land use conversion on microbial communities, it is beneficial to examine both taxonomic and functional responses.

Most research investigating the effects of land use change on soil microbes focuses on taxonomic community responses [6, 52, 66], rather than functional changes. These studies have supported that land use conversion significantly impacts microbial community composition and that these effects endure over time. The few studies examining the functional response of soil communities to land use change have generally sampled a limited number (between three and four) of converted sites [10, 20, 39, 42, 45], with more of an emphasis on assessing temporal replicates. Together, this research indicates that converted sites have distinct functional profiles, with reduced functional diversity generally observed in the converted sites relative to sites retaining the same land use long-term. Winkler et al. [63] estimated that 32% of global land had undergone some form of land use change since 1960, signalling it is increasingly important that we can quantify the long-term impact of these changes on the microbial functions required to sustain vital soil processes.

Soil chemistry, such as organic carbon, does not respond instantly to land use change but reaches a new stability over several years to decades [46]. Therefore, we would expect microbial communities and their functional roles, to respond at a similar rate, due to strong correlations between soil biogeochemistry and microbial community composition [24]. However, more rapid responses by soil microbial communities have been observed, with significant changes seen five years post the conversion event [25]. Additionally, certain practices (e.g. tillage) involved in some land use conversions or land use intensification, can rapidly change soil properties, with declines in soil organic carbon observed after only two years following conversions of prairie grasslands to croplands [51]. To gain a more in-depth understanding of the functional changes occurring after land use conversion, larger-scale studies are required that allow for multiple comparisons between converted sites and their former and current land uses, and at sufficient scale to account for the huge natural spatial variations in bacterial community traits.

This study quantifies the effects of land use conversions on soil bacterial community composition and functional potential. We used shotgun metagenomic and 16S rRNA gene amplicon sequencing to assess soil microbial community functional potential and taxonomic responses to land use change, respectively. We assessed 29 converted sites that had transitioned from grassland to either exotic forest or horticulture and from exotic forest to grassland, comparing them to 38 long-term reference sites representing each land use examined. The converted sites were sampled at differing years following conversion, adding a temporal element to the study. We expect that recently converted sites will harbour microbial communities that resemble their former land uses more closely, since soil physicochemical attributes may take decades to change to reflect their new land use. To test this, we investigated the following questions: (1) Does the composition of soil microbial communities from converted sites more closely reflect their former or current land uses? (2) Are sites converted more recently more like their former land use than those converted earlier? (3) Are trends in the taxonomic response to land use change similarly reflected in changes in functional potential? (4) Are there taxa or functions reproducibly representative of each land use? The answers to these questions are important to better understand the long-term impact of land use change on soil microbial diversity and contributions to global soil health and production potential.

## Methods

### Site description and sample collection

**Fig. 1:**
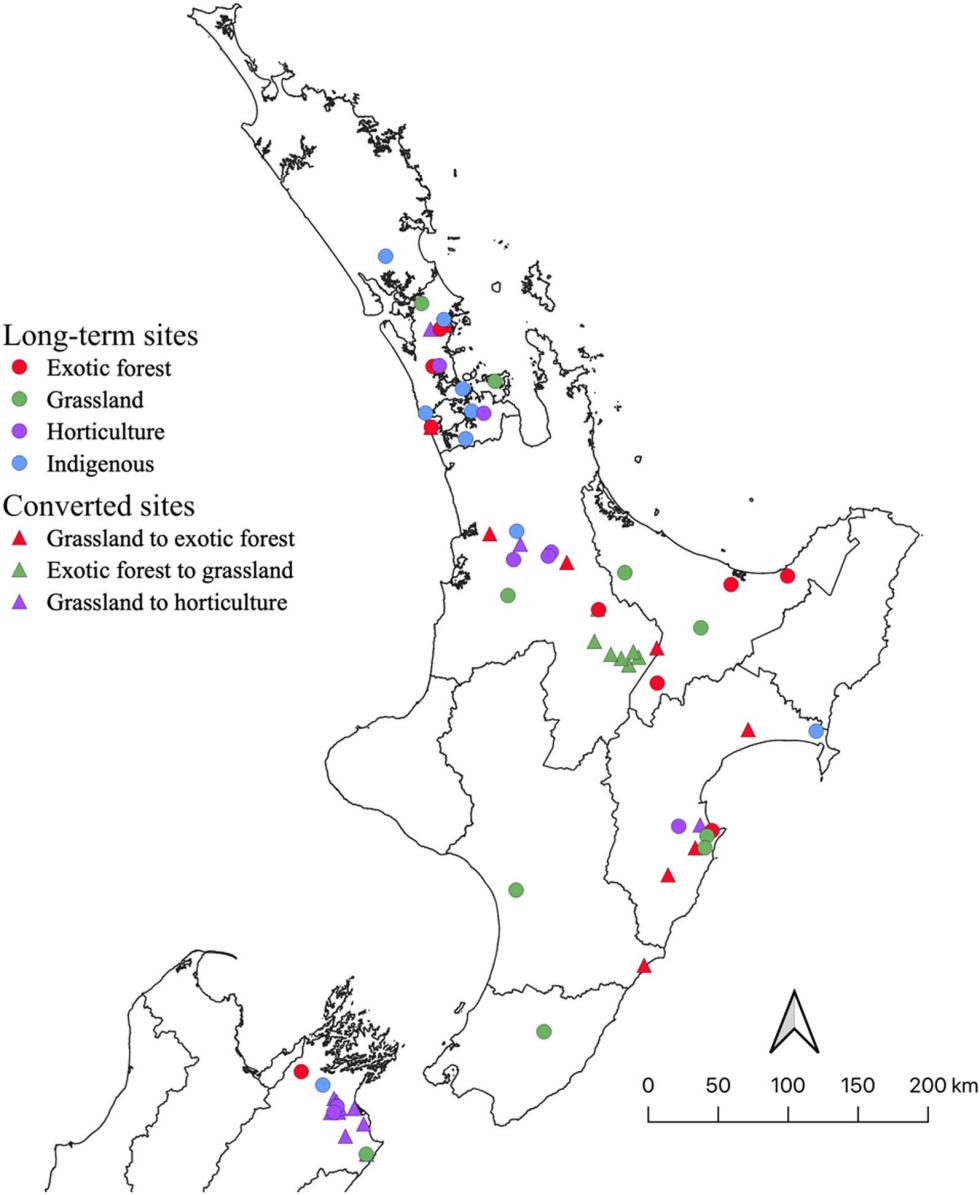
Location of the 67 sites sampled across New Zealand. The points are coloured according to their current land use. Circles identify long-term sites, while triangles represent converted sites

Soil samples were collected from 67 sites (with five replicates per site, *n* = 335) across eight regions of New Zealand between 2013 and 2017 (Fig. 1). Across the sites, mean annual temperature ranged from 10 to 15.7 °C and total precipitation ranged from 567 to 1965 mm per year (Additional file 1: Table S2). The New Zealand Land Cover Database (LCBD version 5.0, January 2020) was used to classify the dominant land use of the sites based on their GPS location (recorded at the start of the sample transect line) and whether they were under their current land use ‘long-term’ or had undergone a land use conversion in the past 20 years. We used the LCDB for classification of the site land use for consistency of classification, reproducibility and potential for higher throughput use for future monitoring. Thirty-eight of the total sites sampled were considered ‘long-term’, having not undergone any significant land use change for at least 20 years. These long-term sites were comprised of exotic forest (*n* = 9), grassland (*n* = 10), horticulture (*n* = 9) and indigenous forest (*n* = 10) dominated land uses. These sites were selected from a previous large-scale project (~ 500 sites), which confirmed that land use significantly correlates to variation in soil microbial community composition across New Zealand [23]. To select sites representative of each land use from this prior dataset and to exclude outlier sites, a non-metric multidimensional scaling (nMDS) ordination was produced based on a Bray–Curtis dissimilarity matrix. The centroid of data representing each land use was calculated and the sites selected in this study were randomly subsampled from those that fell within two standard deviations of the centroid for their corresponding land use (Additional file 1: Fig. S1).

The remaining 29 sites were sampled between two to sixteen years after a land use conversion event. The converted sites consist of areas that have transitioned from exotic forest to grassland (*n* = 7), grassland to exotic forest (*n* = 9) and grassland to horticulture (*n* = 13). The converted sites were classified as either historical or recent conversions based on the length of time since the conversion event and the type of conversion. Exotic forest to grassland sites were classified as historic conversions if sampling took place more than five years after the conversion event, as soil pH and Olsen *P* levels reportedly reach acceptable levels for pasture growth after four years of fertilizer inputs [21] and total carbon and nitrogen reportedly increase most within 1–5 years after conversion from *P. radiata* forest to pasture [50]. Grassland to exotic forest sites were considered historic conversions if the sampling took place more than 11 years after the conversion event, according to findings of [22] which found no significant decline in pH until 11 years after the pine was planted, under various stockings. Grassland to horticulture sites were classified as historic conversions if sampling occurred ten years after the conversion event, following the work of Gentile et al., [18], which suggests the highest fine root turnover and therefore carbon inputs, occur in the first ten years of orchard establishment. We acknowledge there is much variation in horticultural systems due to differences among the crops planted and related differences in cropping and management strategies. However, we chose to treat these diverse systems as one land-use group for the practicality of implementing future monitoring systems for broad land use groups such as horticulture.

We used samples previously collected and described by [24, 23]. In brief, five soil cores (0–10 cm depth, 2.5 cm diameter) were sampled from each site every 10 m along a 50 m transect. A further 25 soil cores were collected every 2 m along each transect and were composited for each site to determine soil physiochemical properties, following national guidelines for soil quality monitoring [27]. For soil physical analyses, three additional stainless-steel rings were used to collect intact soil samples (10 cm depth, 10 cm diameter) every 15 m to measure average bulk density and macroporosity (− 10 kPa).

### DNA extraction, PCR and sequencing

DNA extraction and 16S ribosomal RNA gene amplification were conducted as previously described [23]. In brief, the soil cores were thawed and manually homogenized. Genomic DNA was extracted from 250 mg of each sample using DNeasy PowerSoil HTP kits (Qiagen, Valencia, CA, USA). For the analysis of bacterial community taxonomy, data previously generated were used [24, 23]. The V3-V4 region of 16S rRNA genes was amplified through PCR. Purified PCR products were submitted to New Zealand Genomics Ltd. (Auckland, New Zealand), barcoded (Nextera XT dual indices), pooled and sequenced on an Illumina MiSeq instrument, producing 2 × 300 bp paired-end reads.

New data were generated to analyse the functional potential of the microbial communities at each site. Shotgun metagenomic sequencing was undertaken by Otago Genomics Ltd., New Zealand, to analyse the functional potential of each of the sites. Equal amounts of extracted DNA from each of the site replicates were pooled to produce a composite sample for each site. Sequencing was conducted on an Illumina HiSeq 2500 Instrument, producing sequence lengths of 2 × 150 bp.

### Bioinformatics and statistical analysis

The processing of these sequence data and all downstream analyses were performed in R v 3.6.3 [48]. The Bioconductor pipeline [9] was used to process the demultiplexed 16S rRNA gene reads. We used the DADA2 algorithm to infer amplicon sequence variants (ASVs), determining exact variants at a single-nucleotide level. The fine resolution of DADA2 allows for higher accuracy in determining real biological variants and greater consistency between studies relative to traditional clustering methods [8]. The reads were filtered and trimmed to remove low-quality reads and primers, the paired-end reads merged, and the chimeras were removed using the DADA2 package. Sequence variants were taxonomically classified using the RDP’s naïve Bayesian classifier method [60] and the SILVA rRNA gene database, version 138 [47]. The ASV table, the corresponding taxonomic assignments and the metadata were compiled into a phyloseq object [38]. The ASVs not classified as of bacterial origin were excluded, including sequences classified as being from chloroplasts or mitochondria. As the soil cores for physiochemical analyses were pooled for each site, the site replicate data for microbial analyses were averaged to provide representative bacterial diversity for each of the 67 sites. For alpha-diversity analyses, the reads were normalized through random subsampling without replacement to an even depth of 4875 reads per site (Additional file 1: Fig. S2) using the *rarefy_even_depth* function in the ‘phyloseq’ package [38]. For beta-diversity analyses, we performed Cumulative Sum Scaling (CSS) normalization on the non-rarefied data, after removing all samples with < 1000 reads.

For the processing of the shotgun metagenomic sequences, the quality of the reads was assessed using FastQC v0.11.7 [3], before the trimming of primers and discarding of reads less than 80 bp or those with an average quality score below 30 using Trimmomatic v0.38 [5]. The paired-end reads for each sample were then interleaved into one file for further processing. We used the gene-predicting algorithm Prodigal v2.60 [29] to predict the open reading frames and aligned the sequences against a protein reference database using DIAMOND v0.8.38 [7]. MEGAN6 [28] was used to map the reads to genes with defined functional roles and then into biologically curated SEED subsystems [44]. We then used SEED assignments to assess the general functional profiles of the different samples. The KEGG database (May 2021 version; [32] maps the reads to KEGG Orthology (KO) groups and then to metabolic pathways. We used the KEGG assignments to identify pathways highly representative of land uses. We normalised the SEED and KEGG assignments using CSS for beta-diversity analyses and normalised to the smallest given count to account for read depth variation for alpha-diversity analyses.

### Quantitative analyses

We performed variance partitioning, using the *varpart.MEM* function [35], to determine the relative variance explained by the categories space, soil chemistry, climate and site characteristics on a Bray–Curtis dissimilarity matrix (based on ASVs). The coordinates of each site were used to calculate Moran’s eigenvector maps (MEMs) and those that significantly correlated with the dissimilarity matrix were used as the spatial components in the variance partitioning model. The largest amount of variance explained was by soil chemistry (Additional file 1: Fig. S3). To explore the difference in alpha diversity between microbial communities in the converted sites and their former/present land uses, the Chao1 index and the Inverse Simpson’s index were calculated using the *estimate_richness* function in phyloseq, generating richness and evenness estimates, respectively (based on ASVs or SEED subsystem level 4 assignments, offering the finest functional resolution). We ran a Dunn’s test to identify statistically significant differences among site types. To attain a general representation of the diversity of communities for each of the long-term and converted sites, bar plots were generated that displayed the average (mean) relative abundances of each of the abundant phyla (representing > 1% of relative abundance) and all the subsystem level 1 SEED assignments using the *plot_bar* function in the ‘phyloseq’ package [38]. We applied the Mann–Whitney U test to identify abundant phyla that significantly differ in relative abundance between the respective long-term sites for each conversion type. A Mann–Whitney U test was also used to compare the relative abundance of KEGG level 3 groups within the metabolism category and the abundance of methane (CH_4_) and nitrogen cycling genes, based on level 4 KEGG assignments.

Differences in community composition and functional potential (based on ASVs or SEED subsystem level 4 assignments, respectively) between the communities in converted sites and their corresponding long-term land uses were tested through computing Bray–Curtis dissimilarity matrices and running a permutational multivariate ANOVA (PERMANOVA) [2] applied with the *adonis* function in the ‘vegan’ package [43]. We visualised the dissimilarity matrices using nMDS plots. Environmental factors that significantly correlated with the ordination (*P* < 0.05, calculated using 999 permutations) were fitted onto plots using the *envfit* function in ‘vegan’ [43]. To test the congruence between the shapes of the community composition and functional ordinations, the two ordinations were maximally superimposed during a Procrustes analysis using the function *Procrustes* from the ‘vegan’ package, and a permutational test of significance was run on the Procrustes results, using the function *protest* (based on 999 permutations of these data; [43]. The first axis scores were extracted from both the bacterial community and functional nMDS ordinations to offer a more simplified version of these data to visualise compositional changes over time since conversion events. For each conversion type, the nMDS axis one scores were plotted for the converted sites (separated on the plots as recent or historic conversions) and their respective long-term sites.

Indicator value (IndVal) analyses were conducted separately on the amplicon and KEGG functional data to identify taxa (ASVs) and functions (Level 4 categories, representing the most detailed classification) that were highly representative of the different land uses and each of the converted site types [12]. The function *multipatt* from the R package ‘indicspecies’ was used to run indicator analyses separately on the long-term and the converted site data (with the converted sites split by recent and historic conversions) to identify which indicators were shared [11]. The function ‘euler’ from the R package *eulerr* was used to produce euler diagrams for each converted site type and to identify the number of shared indicators between the historically and recently converted sites and their respective long-term sites [33]. The mean relative abundances of the shared indicator taxa and functions were visualised using stacked bar plots. R scripts for the statistical and quantitative analyses of the functional and taxonomic data are available as Additional files 2 and 3, respectively.

## Results

### Taxonomic and functional diversities across land uses

After quality filtering the amplicon data, we obtained 1.38 million sequencing reads. Relative taxon richness did not significantly differ among the long-term land uses and the only converted site type to significantly differ in richness from their respective long-term land use was the exotic forest to grassland sites, having lower relative ASV richness than their current land use (grassland) (Chao1 index, Dunn’s* P* < 0.05, Additional file 1: Fig. S4a). We acknowledge that the lack of significant differences observed in richness could be due to unmeasured variation, such as site location and the local pedoclimatic conditions. After CSS normalisation, 39,350 unique bacterial ASVs were identified, representing 22 phyla, 232 families and 512 genera. The composition of the long-term horticulture and grassland to horticulture soil communities were significantly more even than the soil communities in the other site types (Inverse Simpson’s index, Dunn’s *P* < 0.05, Additional file 1: Fig. S4b). Largely, the taxonomic richness or evenness of soil microbial communities did not significantly differ between the converted sites and their long-term counterparts (Dunn’s *P* > 0.05, Additional file 1: Fig. S4a, b).

For the shotgun metagenome data, a total of 280 million paired-end 150 bp reads remained after quality filtering, with an average of 4.1 million sequences per sample. Of the reads that could be annotated in MEGAN, using the reference database SEED, the largest percentage of the reads were classified within the ‘metabolism’ category, with an average of 31% of all reads. In contrast, the second-highest category was ‘stress response, defense and virulence’, representing 13% of all reads. Each SEED subsystem level 4 functional category was present in all samples; therefore, the richness at this functional level was consistent across all land use types. Among the long-term sites, the functional data of the horticulture sites were significantly more even than the exotic forest and indigenous sites (Dunn’s* P* < 0.05, Additional file 1: Fig. S4c), with the functional data from the other long-term sites not significantly differing in evenness. The exotic forest to grassland sites were the only converted site type for which the diversity of their subsystem level 4 functions significantly differed from its respective long-term counterparts, being significantly more even, overall than their former land use (exotic forest; Dunn’s *P* < 0.05, Additional file 1: Fig. S4c).

### Microbial taxonomic and functional potential profiles differ between land use types

**Fig. 2:**
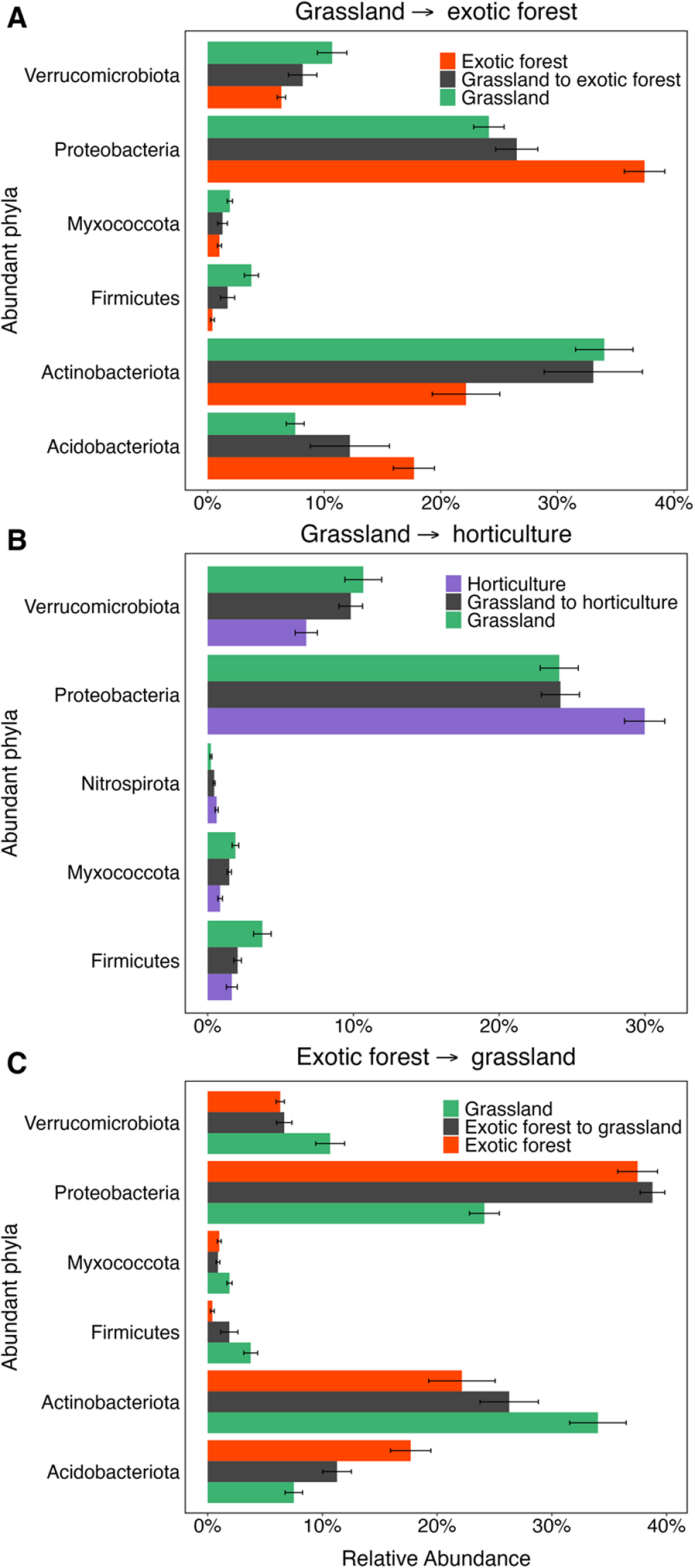
Mean relative abundance of the abundant phyla (representing > 1% of total relative abundance) that significantly differed (Mann Whitney’s U *P* < 0.05) in relative abundance between the respective long-term land use sites for **A** grassland to exotic forest, **B** grassland to horticulture and **C** exotic forest to grassland sites. Error bars represent the standard error

There was more variability in the taxonomic profiles across the different land uses than the functional potential, where no categories significantly differed at functional SEED subsystem level 1 (Mann Whitney U; *P* > 0.05, Additional file 1: Fig. S5). The abundant phyla, classified as making up over 1% of the total relative abundance, were the same phyla for each of the long-term land uses, except for the grassland sites where the phylum *Myxococcota* was also present at > 1% relative abundance, on average. The most abundant phylum across all the long-term land uses was *Proteobacteria*, excluding grassland sites, where *Actinobacteriota* was the most abundant. Within the *Proteobacteria* phylum, *Alphaproteobacteria* was the dominant class in all samples (Additional file 1: Fig. S5a). Six of the abundant phyla significantly differed in relative abundance between the long-term exotic forest and grassland sites, with exotic forests presenting greater relative abundances of *Proteobacteria* and *Acidobacteriota* and grassland sites presenting greater levels of *Verrucomicrobiota*, *Myxococcota, Firmicutes* and *Actinobacteriota* (Fig. 2a and c Mann Whitney U *P* < 0.05). The relative abundance of these phyla in the exotic forest to grassland and the grassland to exotic forest conversion sites were in between their abundances in both the long-term sites, except for *Myxococcota* and *Proteobacteria* in the exotic forest to grassland converted sites, which was less abundant in converted site than both long-term sites and more abundant in the converted site than the long-term sites respectively. Five of the dominant phyla significantly differed in relative abundance between the long-term grassland and horticulture sites, with *Verrucomicrobiota, Myxococcota* and *Firmicutes* present at higher relative abundance in the long-term grassland sites and *Proteobacteria* and *Nitrospirota* more abundant in the long-term horticulture sites (Fig. 2b Mann Whitney U *P* < 0.05). The same trend of convergence towards their current long-term land use was also seen for the grassland to horticulture sites.

**Fig. 3:**
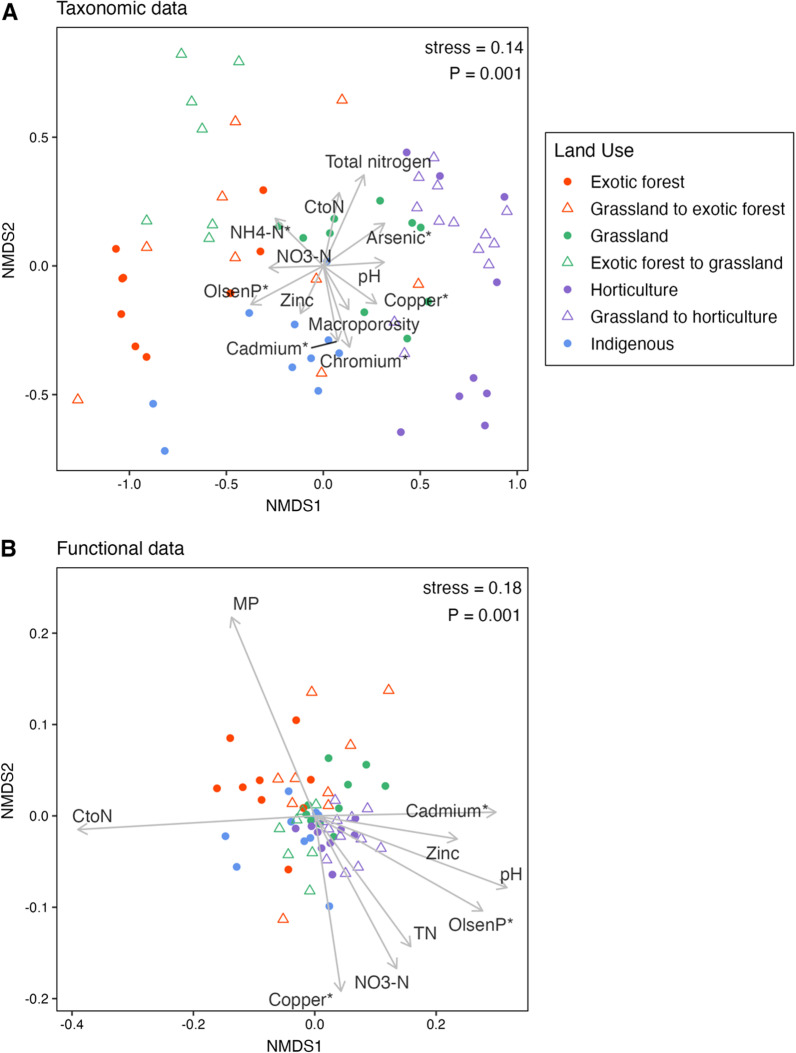
Bray–Curtis dissimilarity based non-metric multidimensional scaling (nMDS) ordinations of **A** bacterial community composition (based on ASVs) and **B** community functional potential (based on SEED subsystem level 4 categories). Environmental variables that significantly correlated (*P* < 0.05 based on 999 permutations) with the ordinations are represented as vectors on the plots. nMDS stress values and *P*-values from PERMANOVA assessing site-type effects are displayed in the top right corner

The taxonomic composition and functional potential of the soil bacterial communities clustered according to their land use type (Fig. 3). A Procrustes rotation analysis revealed the congruence of the taxonomic and functional ordinations, indicating a significant correlation between them (*r* = 0.66, *P* < 0.001). Community composition and functional composition significantly differed (*P* < 0.001 for all PERMANOVA pairwise comparisons) between each site type: the four long-term land uses and the three converted site types (grassland to exotic forest, grassland to horticulture, exotic forest to grassland). Visually, the taxonomic ordinations appear to display more distinct clustering-based on land use type than the functional ordination.

### Microbial communities from converted sites respond in an expected manner

**Fig. 4:**
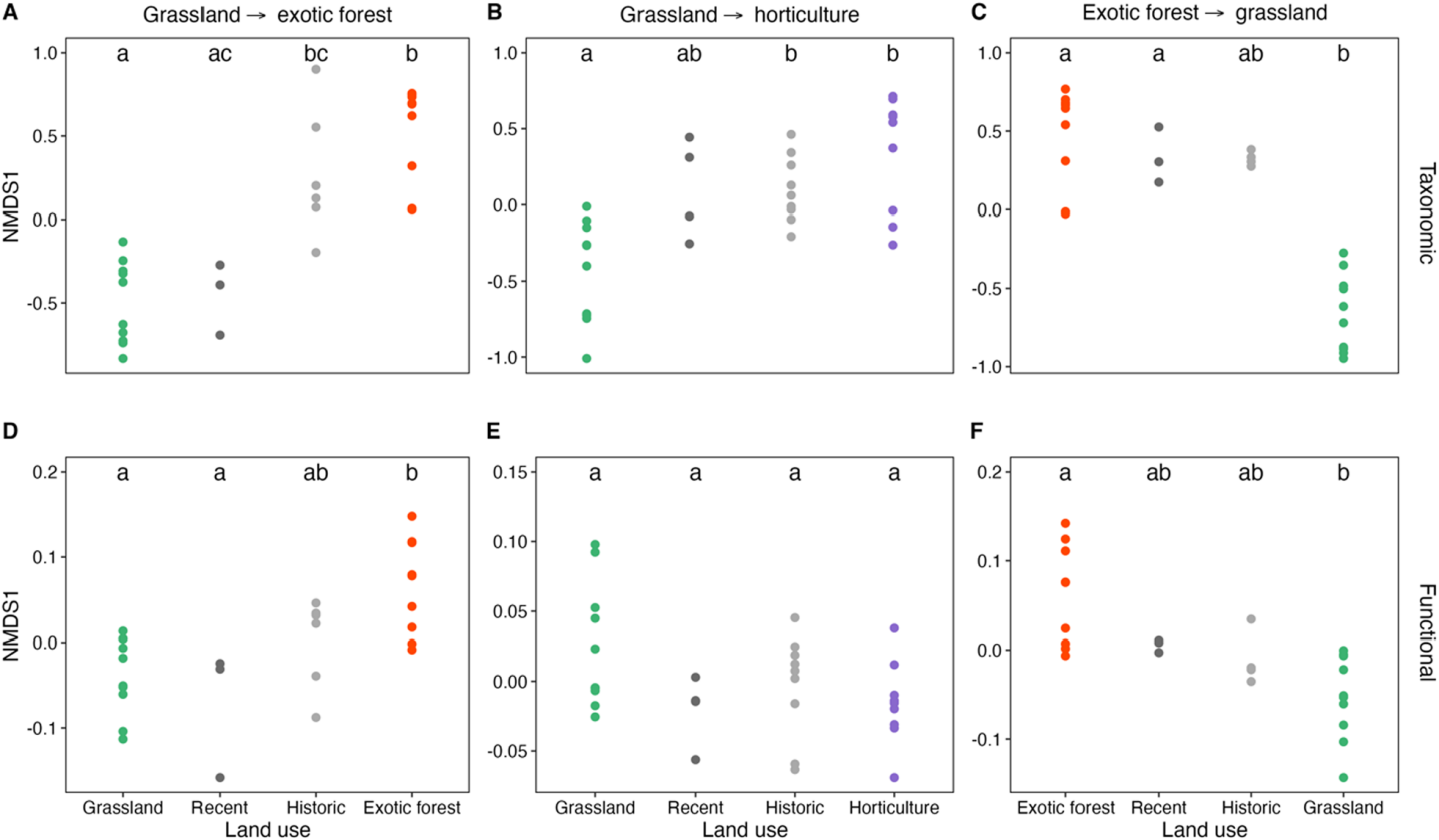
Axis 1 scores of Bray–Curtis based nMDS ordinations of **A**–**C** bacterial community composition (based on relative abundances of ASVs) and **D**–**F** functional potential (based on SEED subsystem level 4 categories) for the three conversion types: **A** and **D** grassland to exotic forest, **B** and **E** grassland to horticulture and **C** and **F** exotic forest to grassland. Each plot displays the nMDS scores of the primary axis for the respective long-term land use site and the converted sites, split by whether they are classified as recent or historical conversions. Boxes with different letters within each panel indicate significant differences from each other (Dunn’s *P* < 0.05)

For a simplified representation of these data, we extracted the first axis data scores of the nMDS ordinations of the taxonomic profiles and functional potential for each converted site type and their respective long-term land use sites (Fig. 4). We separated sample data from the converted sites based on whether they were historic or recent conversions. Procrustes analyses indicated a significant correlation between each taxonomic and functional single-axis ordination per conversion type (Procrustes, exotic to grassland (*r* = 0.76, *P* < 0.001), grassland to exotic (*r* = 0.80, *P* < 0.001), grassland to horticulture, (*r* = 0.37, *P* < 0.02). No significant differences were observed between the historic or recently converted samples for any of the three conversion types (each Dunn’s *P* > 0.05). For the taxonomic data, we found sites that were converted more recently had greater similarities to their former long-term land uses. Recently converted samples for both the exotic-to-grassland and grassland-to-exotic forest converted sites significantly resembled their former land use (Dunn’s *P* < 0.05) and not their current long-term land use (Dunn’s *P* > 0.05). Soil communities under historic land use conversions were more like their current long-term land use, with data from historically converted grassland to horticulture sites and grassland to exotic sites being significantly like those representing their current land use (Dunn’s *P* < 0.05), and not their former (Dunn’s *P* > 0.05). The gradual convergence of community data to their current long-term land use occurs for each conversion site type. A comparable but less pronounced trend was seen in the functional potential ordinations, excluding the grassland to horticulture samples. No significant difference was detected among data from each of the four land use categories (Dunn’s *P* > 0.05).

### Taxa and functions are representative of land use

**Fig. 5:**
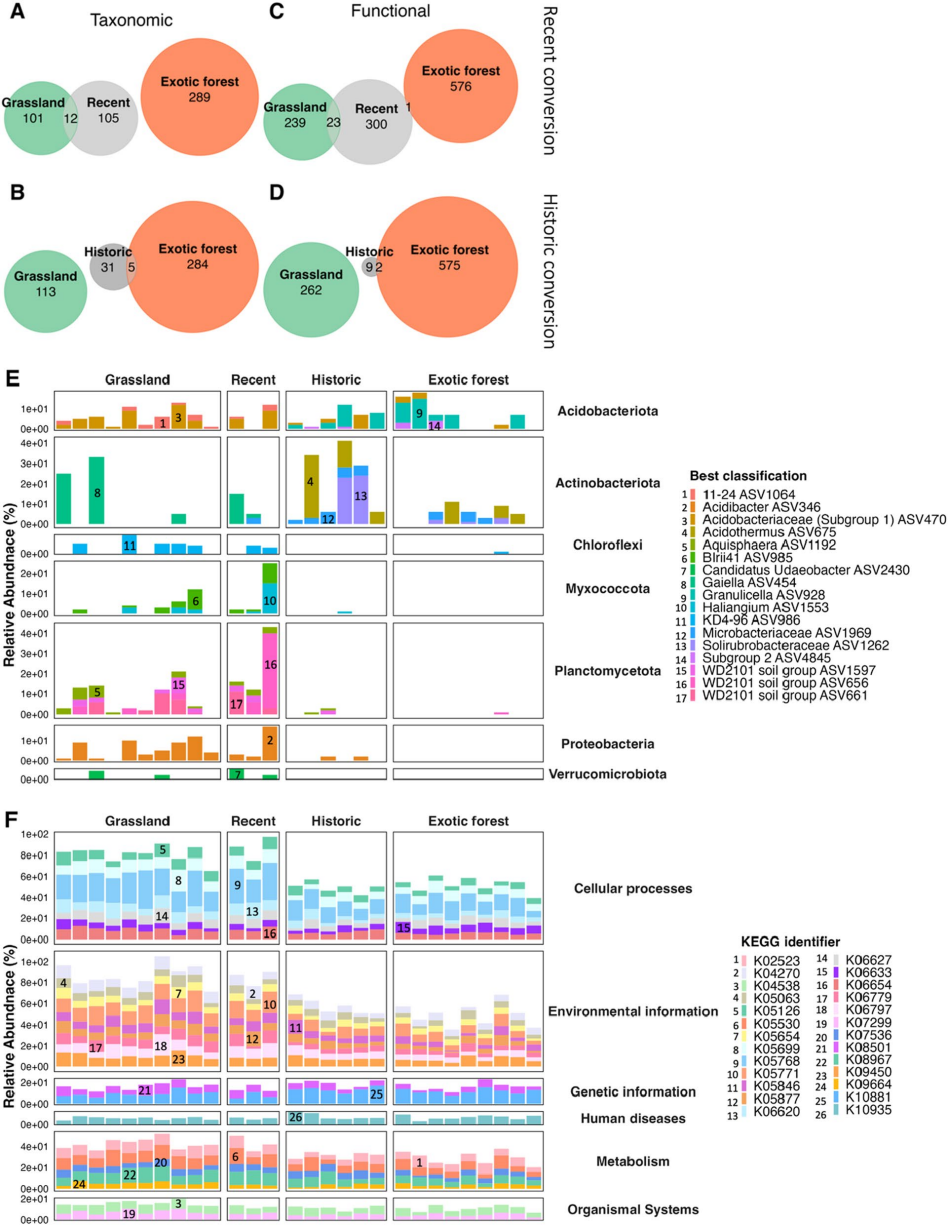
Euler diagrams indicating the observed number of **A** and **B** shared indicator ASVs and **C** and **D** indicator functional groups (based on Level 4 KEGG categories) between the **A** and **C** recently converted and **B** and **D** historically converted grassland to exotic forest sites and their former (grassland) and current (exotic forest) long-term land use sites. Stacked bar charts visualizing the mean relative abundance of the shared indicator ASVs, split by **E** the different phyla the taxa are assigned to and **F** the indicator functional groups, split by the Level 1 KEGG categories for the grassland to exotic forest sites and their long-term sites. The numbers in the legends correspond to the numbers on the bars. For comparisons between grassland to horticulture and exotic forest to grassland conversions, see Additional file 1: Figs. S6&S7

An indicator species analysis (IndVal), conducted separately on the long-term and converted site data, identified taxa (ASVs) and functional categories (KEGG level 4 categories) that were significantly associated with the different site types. In the taxonomic data, the recently converted sites only shared indicators with their former land use sites (Fig. 5 and Additional file 1: Fig. S6). The historic conversion sites only shared indicators with their current land use sites, excluding the historic exotic to grassland sites which shared one indicator with the long-term exotic sites (Fig. 5 and Additional file 1: Fig. S6). The functional data displayed similar but less defined patterns, as was expected given the stronger distinction between site types in the taxonomic data. The recently converted sites shared most indicator functional genes with their former land use, excluding horticulture to grassland sites, which shared two indicators with the grassland sites representing their prior land use and four with the horticulture sites representing their current land use. The historic conversions only shared indicators with their current land use, excluding the exotic forest to grassland sites. They shared 15 functional indicators with the exotic sites representing their prior land use and one with the grassland sites representing their current land use (Additional file 1: Fig. S6h).

Out of the 12 indicator taxa shared between the long-term grassland sites and the recently converted grassland to exotic forest sites, only five were identified in at least one of the historically converted sites (Fig. 5e). Out of the five indicator taxa identified in both the long-term exotic forest sites and historically converted grassland to exotic forest sites, only one taxon was observed in the recently converted sites (Fig. 5e). Most of the indicator taxa from the former land use were only observed in the recently converted sites, and most of the taxa from the current land use were only identified in the historically converted sites. We also observed a shift in the relative abundance of indicator taxa in the sample data of the converted sites from resembling their former land use to their current land use through time since land use conversion. A similar trend was observed for the other two conversion types (Additional file 1: Fig. S7a and b). Each of the shared indicator functional categories was observed in the converted sites and their respective long-term land use sites for each conversion type. However, the relative abundance of the shared indicator functional categories from the recently converted sites generally resembled their former land use. In contrast, the relative abundance of the functional genes from historic conversion sites resembled their current land use (Fig. 5f and Additional file 1: Fig. S7c and d).

### The abundance of functional categories involved in metabolism pathways differ with land use

**Fig. 6:**
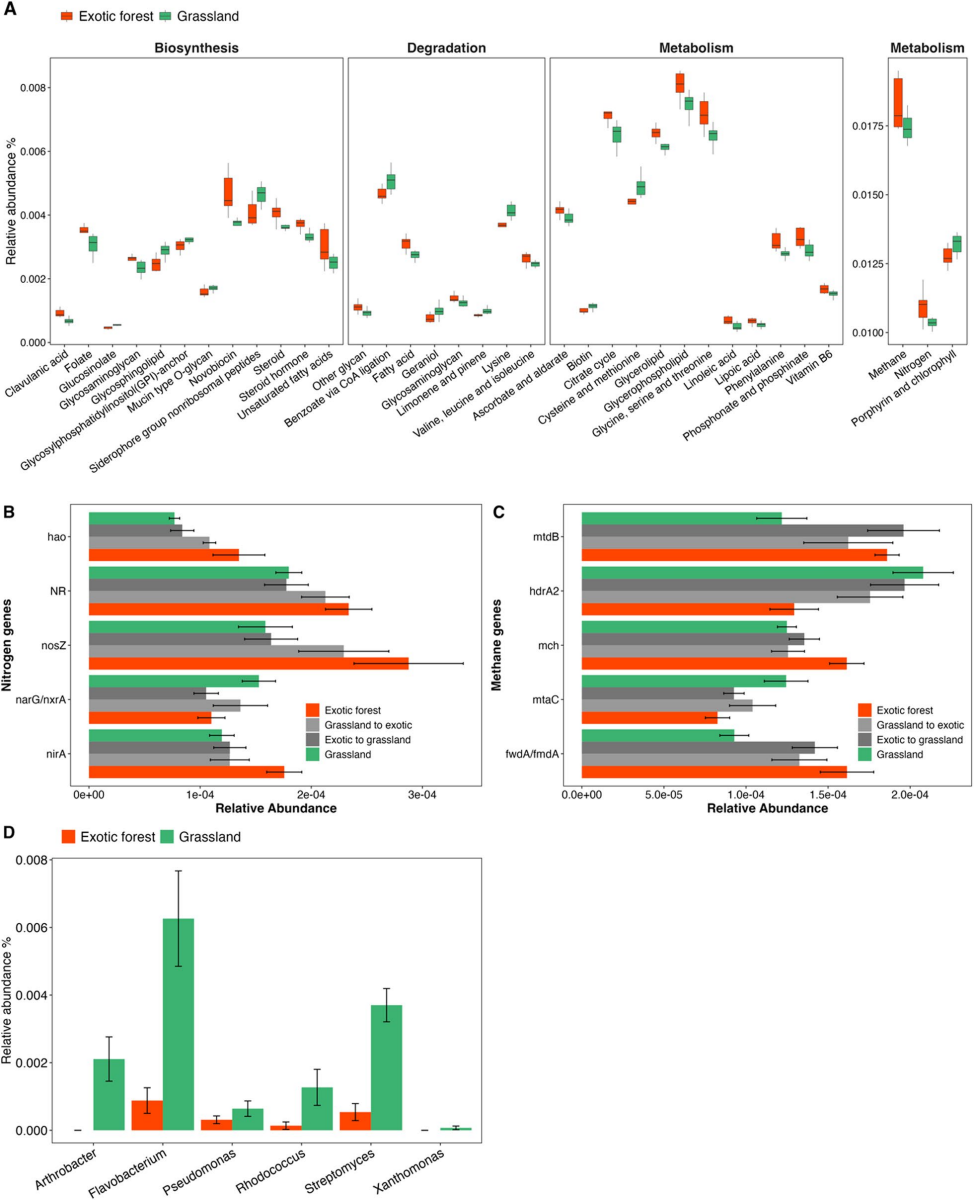
**A** Boxplots display the relative abundance of the level 3 KEGG pathway categories, within the level 3 category metabolism, which significantly differed in relative abundance between the long-term exotic forest and grassland sites (Mann–Whitney’s U *P* < 0.05). The categories are split by whether they relate to biosynthesis, degradation or metabolism. The metabolism category is split by relative abundance with categories with > 0.01 displayed in the box on the right. The boxes represent the interquartile range (IQR: 25–75% of the data), the horizontal line indicates the median, while the whiskers extend to 1.5 times the IQR. Mean relative abundance of level 4 **B** nitrogen and **C** methane cycling genes and **D** aromatic compound, lysine and geraniol degraders that significantly differed (Mann Whitney’s U *P* < 0.05) in relative abundance between the long-term grassland sites and the long-term exotic forest sites. Error bars represent the standard error

We focused on metabolism pathways due to the important role the soil microbial community has in metabolic processes, such as nutrient cycling. Thirty-five metabolism level 3 KEGG categories significantly differed in relative abundance between the long-term grassland and exotic forest sites (Fig. 6a, Mann–Whitney U *P* < 0.05). Twenty-three of the metabolism-related pathways were more abundant in the exotic forest sites, while twelve were more abundant in the grassland sites. Within the degradation category, genes encoding for benzoate degradation via CoA ligation were significantly more abundant in the grassland sites. This pathway involves the degradation of molecules containing aromatic rings (e.g., benzene, toluene). In the taxonomic data, bacterial genera able to degrade aromatic compounds such as *Pseudomonas, Rhodococcus, Streptomyces, Arthrobacter* and *Xanthomonas* were more abundant in the grassland sites (Fig. 6d). Lysine and geraniol degradation-related genes were also significantly more abundant in the grassland soils (Fig. 6a). Taxa in the genus *Pseudomonas* are capable of degrading both lysine and geraniol. Additionally, taxa within the genus *Flavobacterium* can degrade geraniol and were significantly more abundant in the grassland sites (Fig. 6d).

The relative abundance of genes encoding for methane and nitrogen metabolism were significantly higher in mean relative abundance in the exotic forest sites, relative to the grassland sites (Fig. 6a). We further investigated the level 4 KEGG categories of nitrogen and methane metabolism due to the important role the microbial community plays in nitrogen and methane cycling. Five key nitrogen cycling genes significantly differed in relative abundance between the long-term grassland and exotic forest sites, with one gene (*narG*), involved in reduction of nitrate to nitrite, having higher relative abundance in the grassland sites and four (*hao, NR, nosZ, nirA*) in the exotic forest sites (Fig. 6b Mann Whitney U *P* < 0.05). These four genes are involved in the oxidation of hydroxylamine to nitrite, reduction of nitrate to nitrite, reduction of nitrous oxide to nitrogen and reduction of nitrite to ammonia respectively, The relative abundance of the genes in the converted sites were intermediate to their abundances in both the long-term sites for all five genes, except *NR* and *NarG,* which had lower relative abundance in the exotic forest to grassland sites. Five methanogenic genes significantly differed in relative abundance between the long-term grassland and exotic forest sites, with two genes (*hdrA2, mtaC*) having higher relative abundance in the grassland sites and three (*mtdB, mch, fwdA*) in the exotic forest sites (Fig. 6c Mann Whitney U *P* < 0.05). These genes are all involved in methanogenesis and more specifically, in the reduction of coenzyme M to coenzyme B, methyl transfer from methanol to coenzyme M to form Methyl-CoM, the oxidation of methylene-dH_4_MTP to methenyl-dH_4_MTP, conversion of methenyl-dH_4_MTP to formyl-dH_4_MTP and the reduction of CO_2_ to N-formylmethanofuran respectively. The relative abundance of the genes in the grassland to exotic forest and the exotic forest to grassland sites were intermediate to their abundances in both the long-term sites for all five genes, except *mtdB,* which had higher relative abundance in the exotic forest to grassland sites.

## Discussion

It is important to understand the long-term consequences of land use changes on the potential for soil microbes to undertake and sustain key functions of relevance for soil health, production potential and global processes. Our results indicate that although the communities within the converted sites were compositionally distinct from the communities in their former and current land uses, there was a shift from communities in recently converted sites more closely reflecting the composition of their former land use, to communities from sites with older conversion events being more compositionally like their current land use. Importantly, we also saw this trend in the functional potential of the converted sites, indicating that the lasting impacts of historic land use legacy extend to the functioning of microbial communities, including critical processes.

### Over time, taxonomic composition in converted sites converges to resemble bacterial composition representative of current land uses

In line with various studies [31, 25, 45, 66], we observed that land use change results in alterations to the composition and functional potential of soil microbial communities. Our study adds to the current knowledge by providing insights into the time frame that these changes occur, with bacterial communities within grassland to exotic forest and grassland to horticulture-converted sites already compositionally distinct from their former land use and compositionally comparable to their ‘future’ land use within 11 and 10 years, respectively (Fig. 4a and b). Changes to microbial communities or biomass within a similar time frame have been observed in sites converted from grassland to *Eucalyptus* forest [49] and over a longer time frame (between 19 and 160 years) in grassland to cropland sites across Europe [54]. However, these prior studies identified alterations to bacterial communities after land use change, rather than measuring whether directional changes in the bacterial community composition occurred. We observed directional compositional changes through time; dominant phyla shifted in relative abundance towards resembling their abundances in soils under their current land use category.

Although still compositionally like their former land use, sites converted from exotic forest to grassland began to resemble their current land use more closely after only five years (Fig. 4c). This was confirmed as the bacterial community composition in the historically converted sites was compositionally comparable to their ‘current’ land use, and a directional shift was observed in the relative abundance of dominant phyla. These results are supported by a previous study, where the taxonomic and functional compositions of sites converted from pine forest to pasture were more similar to pasture than to pine forest communities after five years and progressively similar to long-term pasture communities, eight years post-conversion [25]. This current study builds on this prior research by identifying similar trends across a larger dataset with various land use conversion types. Such findings of a gradual convergence in taxonomic composition and functional potential from former to current land use, demonstrate the extent that we can predict microbial community taxonomic and functional change, based on the length of time since land conversion events. This is important for improving land management and conservation decisions, as it indicates that bacterial communities can be useful in assessing soil conditions after environmental disturbance.

### Taxonomic compositional changes of soil bacterial communities to land use alteration are reflected in functional responses

Changes in bacterial community composition associated with land use conversion translated to changes observed in the functional potential of the soil communities. The most marked response of the functional composition shifting to reflect the current land use over time was within the grassland to exotic forest sites, which accompanies the most defined taxonomic shift (Fig. 4a). However, for sites converted in the opposite direction, from exotic forest to grassland, the overall composition of functional potential in the converted sites was comparable to the composition within both the respective long-term land uses (Fig. 4f). This response was slightly less defined than that observed in the taxonomic data. Broadly, these results emphasise land use change does impact the functional potential of soil environments. However, we did not observe significant differences in functional composition between the long-term grassland and horticulture sites, or those converted from grassland to horticulture, even though there were taxonomic differences between the long-term sites and a taxonomic shift in the grassland to horticulture sites (Fig. 4e). Therefore, we cannot rule out the occurrence of functional redundancy in these site types. As diversity is often regarded as the dominant factor influencing the functioning of ecosystems, increasing efforts are focusing towards understanding the relationship between microbial biodiversity and soil functioning [58], with various studies linking microbial diversity to maintaining functional processes [39] and functional stability [19, 55]. Additionally, it has been shown that high levels of initial community evenness can protect functional stability under stressed conditions [64]. Although the soil environments in the current study generally maintained their ASV richness after land use conversion, the taxonomic data in the long-term horticulture and grassland to horticulture sites were more even than the other site types. Heightened evenness could potentially increase the likelihood of stress-tolerant species in these communities relative to a community with low evenness. This, in turn, may maintain functional stability, explaining why a shift in functional potential did not accompany the observed taxonomic shift after the land use change from grassland to horticulture. Soil communities in horticulture sites or sites that have transitioned to horticulture might be more resilient to environmental disturbances than the other site types.

### Taxa and functional genes highly representative of different land uses remain at elevated abundances in converted landscapes

We identified taxa and functional genes highly representative of the different long-term land uses, indicating strong associations of key individuals and processes to land use, in line with previous studies [24, 40, 45]. Examining how these representative taxa or functional genes respond to land use change can indicate the degree to which conversions can impact the abundance of ecologically important bacteria and functional groups. Our results suggest the relative abundances of these representative taxa and functional genes are strongly influenced by the process of land use change, with most taxa and functional genes defined as ecologically important in the long-term sites, not being associated with the converted sites (Fig. 5 and Additional file 1: Fig. S6). This suggests that although we observed directional compositional changes in the soil microbial communities of the converted sites after 5–11 years, a longer period post-conversion may be required to establish these key groups as significantly abundant in the converted sites. However, the taxa and functional genes that remained at elevated abundances in the converted landscapes did change predictably, based on the length of time since land use conversion, as we observed a shift of sharing more indicators with their former land use to their current, and a shift in the relative abundance towards resembling the current long-term land use. These results, supported by previous research [23], indicate that monitoring changes in certain key taxa and functional genes after land use change can provide biologically relevant information for soil health monitoring.

### Long-term consequences of land use change on the potential for microbes to undertake key processes for soil health

By examining the responses of different functional groups to land use change, we can establish related long-term impacts on essential processes and, in turn, for soil health. Benzoate is a common active ingredient in agricultural pesticides applied in New Zealand. Correspondingly, the KEGG pathway for benzoate degradation via CoA ligation and taxa capable of degrading aromatic compounds such as benzene, had significantly higher mean relative abundance in the grassland sites than the exotic forest sites (Fig. 6). Aromatic compound degradation is linked with organic carbon turnover through the release of carbon dioxide [17]. Therefore, heightened degradation of aromatic compounds in the grassland sites could indicate lower soil carbon stability and in turn impact the soil’s ability to mitigate climate change [37]. Additionally, the relative abundance of the pathway for degradation of the volatile organic compound (VOC) geraniol was higher in the grassland sites, relative to the exotic forest sites. It has been reported that VOC metabolism may produce substrates that can be used by bacteria capable of degrading biphenyl (a benzenoid aromatic compound), in turn enhancing the degradation of polychlorinated biphenyls and polycyclic aromatic hydrocarbons [26]. As this study demonstrates that land use legacy effects can last for up to a decade, it emphasises the urgency of swiftly implementing strategies to limit loses of soil carbon.

Lysine is often a limited amino acid in the diet of dairy cows and lysine supplemented feed is used to address this deficiency [13]. This may be why we observed higher relative abundances of lysine degradation genes in grassland sites, predominantly used for intensive grazing. *Pseudomonas aeruginosa* is capable of degrading lysine [16] and had higher relative abundance in the grassland sites (Fig. 6d). This suggests that the presence of certain genes and functional pathways could offer the potential to differentiate between land uses or the legacy effects of land use change.

Our data indicates that land use did impact nitrogen and methane cycling, as both pathways were more abundant in the exotic forest sites than the grassland sites, while the relative abundances of the key cycling genes in the converted sites were typically in between those in the long-term sites (Fig. 6b and c). This suggests that like the shift we observed in our taxonomic data, directional changes in the relative abundance of key nutrient cycling genes occur as they shift towards resembling their abundances under their current land use category. Consequently, analysis of the prevalence of microbial functional genes may offer a more holistic measure of soil health than only relying on the analysis of abiotic soil characteristics. This would be further strengthened by measuring rates of processes in situ, for example, nitrogen fluxes or degradation of specific carbon sources. Understanding the intricacies of nutrient regulation in soils impacted by anthropogenic activity is vital to improve our understanding of what drives environmental changes.

## Conclusion

Our study demonstrates that both bacterial community composition and functional potential are shaped by land use. When land use conversion occurs, the microbial communities respond in a somewhat predictable manner to the associated changes in soil conditions. This research will better improve our understanding of the lasting impact of land use on soil microbial communities and how this translates to the functional gene expression of complex microbial communities. Discerning the sensitivity and responsiveness of microbial communities to land use change will assist us in applying biotic variables to soil health monitoring techniques for a more well-rounded approach to the sustainable management of our soil environments.

## Supplementary Information

**Additional file 1.** Supplementary information.

**Additional file 2.** R code associated with functional data processing.

**Additional file 3.** R code associated with taxonomic data processing.

## Data Availability

The amplicon sequence data are available in the NCBI Sequence Read Archive under the following accession numbers, PRJNA578562 and PRJNA323375. All metagenomic sequence data are available in the NCBI Sequence Read Archive under accession number PRJNA825700.
