# Supplementary material for: Land use modification causes slow, but predictable, change in soil microbial community composition and functional potential
Source: Environ Microbiome. 2023 Apr 6;18:30. doi: 10.1186/s40793-023-00485-x (PMC10080853; doi:10.1186/s40793-023-00485-x)
Supplement: Supplementary file 1 — Additional file 1. Supplementary information. [file 40793_2023_485_MOESM1_ESM.docx]

**Supplementary figures:**

Table S1: Land use classification for sites sampled.

| **Land use** | **Description** |
| --- | --- |
| **Exotic forest** | Principally exotic pine forests established largely for forestry production. |
| **Grassland** | High producing exotic grass-legume pastures used for intensive grazing. |
| **Horticulture** | Either orchards, vineyards or other perennial crops. Occasional cultivation is undertaken for weed control. |
| **Indigenous** | A combination of broadleaved or beech forests, with areas of successional communities such as scrub and mānuka and/or kanuka. |

*Adapted from Landcare Research New Zealand Ltd. 2020. New Zealand Land Cover Database (LCDB) classes at version 5.

Table S2: Climatic data (mannt (mean annual temperature (°C)) , Jan maxt (January maximum temperature (°C)), July mint (July minimum temperature (°C)), Rain days (annual rain days), tot precip (total precipitation (mm)), sum precip (summer precipitation (mm)), wint precip (winter precipitation (°C))) for each sampling location. The data are from NIWA interpolations of climate station data and extracted using GIS software (Wratt *et al.*, 2006).

| **Land use** | **Mannt** | **Jan maxt** | **July mint** | **Rain days** | **Tot precip** | **Sum precip** | **Wint precip** |
| --- | --- | --- | --- | --- | --- | --- | --- |
| Exotic forest | 15.1 | 23.0 | 7.7 | 136 | 1089 | 210 | 369 |
| Exotic forest | 14.5 | 22.9 | 6.1 | 143 | 1408 | 280 | 454 |
| Exotic forest | 14.3 | 23.2 | 5.1 | 143 | 1204 | 233 | 409 |
| Exotic forest | 10.0 | 21.7 | -0.2 | 130 | 1380 | 334 | 384 |
| Exotic forest | 12.6 | 23.7 | 2.6 | 125 | 1457 | 322 | 431 |
| Exotic forest | 12.3 | 21.4 | 3.6 | 118 | 1739 | 361 | 519 |
| Exotic forest | 13.1 | 23.6 | 2.9 | 93 | 606 | 107 | 226 |
| Exotic forest | 12.9 | 24.2 | 1.7 | 78 | 582 | 125 | 171 |
| Exotic forest | 12.9 | 24.2 | 1.7 | 78 | 582 | 125 | 171 |
| Grassland | 15.0 | 23.5 | 6.7 | 127 | 1139 | 207 | 384 |
| Grassland | 14.7 | 23.0 | 6.4 | 132 | 1070 | 188 | 366 |
| Grassland | 15.1 | 23.3 | 7.1 | 132 | 1251 | 241 | 406 |
| Grassland | 12.9 | 24.1 | 1.5 | 110 | 1205 | 294 | 342 |
| Grassland | 12.2 | 22.0 | 3.3 | 136 | 1965 | 428 | 593 |
| Grassland | 12.6 | 24.0 | 2.7 | 122 | 790 | 137 | 258 |
| Grassland | 13.1 | 23.9 | 2.7 | 92 | 625 | 114 | 230 |
| Grassland | 12.5 | 23.3 | 2.6 | 99 | 1003 | 197 | 336 |
| Grassland | 12.9 | 22.3 | 4.2 | 125 | 967 | 216 | 274 |
| Grassland | 12.9 | 22.0 | 4.5 | 72 | 594 | 128 | 185 |
| Horticulture | 14.8 | 23.4 | 5.7 | 140 | 1172 | 226 | 397 |
| Horticulture | 14.5 | 24.2 | 4.9 | 138 | 1195 | 213 | 398 |
| Horticulture | 13.5 | 24.7 | 3.5 | 90 | 608 | 140 | 201 |
| Horticulture | 12.8 | 24.0 | 1.8 | 82 | 652 | 141 | 193 |
| Horticulture | 12.8 | 24.0 | 1.8 | 82 | 652 | 141 | 193 |
| Horticulture | 12.4 | 24.1 | 1.7 | 78 | 601 | 131 | 168 |
| Horticulture | 12.9 | 24.2 | 1.7 | 78 | 582 | 125 | 171 |
| Horticulture | 12.7 | 24.2 | 1.6 | 80 | 589 | 127 | 172 |
| Horticulture | 12.7 | 23.0 | 3.2 | 78 | 590 | 123 | 180 |
| Indigenous | 15.1 | 23.2 | 7.9 | 136 | 1272 | 251 | 423 |
| Indigenous | 14.7 | 23.4 | 6.4 | 145 | 1194 | 237 | 387 |
| Indigenous | 14.8 | 23.2 | 6.4 | 143 | 1344 | 268 | 440 |
| Indigenous | 14.7 | 23.4 | 6.4 | 145 | 1194 | 237 | 387 |
| Indigenous | 15.7 | 23.5 | 7.7 | 133 | 1081 | 189 | 366 |
| Indigenous | 14.7 | 23.5 | 6.4 | 139 | 1073 | 196 | 352 |
| Indigenous | 13.8 | 22.8 | 5.6 | 124 | 1314 | 197 | 467 |
| Indigenous | 12.7 | 23.9 | 1.8 | 86 | 826 | 180 | 241 |
| Indigenous | 11.9 | 22.7 | 0.9 | 108 | 1362 | 297 | 352 |
| Indigenous | 15.3 | 24.0 | 7.3 | 143 | 1190 | 234 | 378 |
| Ex to grass | 12.8 | 23.6 | 2.7 | 77 | 567 | 114 | 176 |
| Ex to grass | 12.7 | 23.9 | 1.8 | 86 | 826 | 180 | 241 |
| Ex to grass | 11.9 | 22.7 | 0.9 | 108 | 1362 | 297 | 352 |
| Ex to grass | 12.8 | 24.1 | 1.6 | 80 | 592 | 129 | 173 |
| Ex to grass | 12.7 | 24.2 | 1.6 | 80 | 589 | 127 | 172 |
| Ex to grass | 12.7 | 23.0 | 3.2 | 78 | 590 | 123 | 180 |
| Ex to grass | 11.9 | 22.2 | 1.9 | 137 | 1466 | 347 | 425 |
| Grass to ex | 15.0 | 22.9 | 7.6 | 137 | 1094 | 211 | 372 |
| Grass to ex | 14.7 | 23.1 | 6.4 | 143 | 1360 | 269 | 442 |
| Grass to ex | 12.8 | 24.0 | 2.7 | 91 | 747 | 146 | 260 |
| Grass to ex | 12.6 | 24.3 | 2.9 | 102 | 663 | 137 | 230 |
| Grass to ex | 13.0 | 23.4 | 4.0 | 127 | 1250 | 231 | 369 |
| Grass to ex | 12.5 | 21.3 | 4.7 | 124 | 974 | 164 | 334 |
| Grass to ex | 12.1 | 23.2 | 1.6 | 80 | 624 | 130 | 178 |
| Grass to ex | 12.1 | 23.2 | 1.6 | 80 | 624 | 130 | 178 |
| Grass to ex | 12.9 | 22.0 | 4.5 | 72 | 594 | 128 | 185 |
| Grass to hort | 14.6 | 23.1 | 6.1 | 139 | 1392 | 274 | 450 |
| Grass to hort | 13.6 | 24.3 | 3.2 | 87 | 600 | 117 | 213 |
| Grass to hort | 12.8 | 24.1 | 1.6 | 80 | 592 | 129 | 173 |
| Grass to hort | 12.3 | 24.0 | 1.5 | 79 | 624 | 136 | 173 |
| Grass to hort | 12.7 | 24.3 | 1.7 | 76 | 578 | 124 | 166 |
| Grass to hort | 12.9 | 22.0 | 4.5 | 72 | 594 | 128 | 185 |
| Grass to hort | 12.6 | 23.4 | 1.8 | 102 | 1361 | 291 | 388 |
| Grass to hort | 12.6 | 23.4 | 1.8 | 102 | 1361 | 291 | 388 |
| Grass to hort | 12.4 | 24.1 | 1.7 | 78 | 601 | 131 | 168 |
| Grass to hort | 12.8 | 23.6 | 2.7 | 77 | 567 | 114 | 176 |
| Grass to hort | 12.7 | 24.3 | 1.7 | 76 | 578 | 124 | 166 |
| Grass to hort | 12.3 | 24.0 | 1.5 | 79 | 624 | 136 | 173 |
| Grass to hort | 12.9 | 22.0 | 4.5 | 72 | 594 | 128 | 185 |


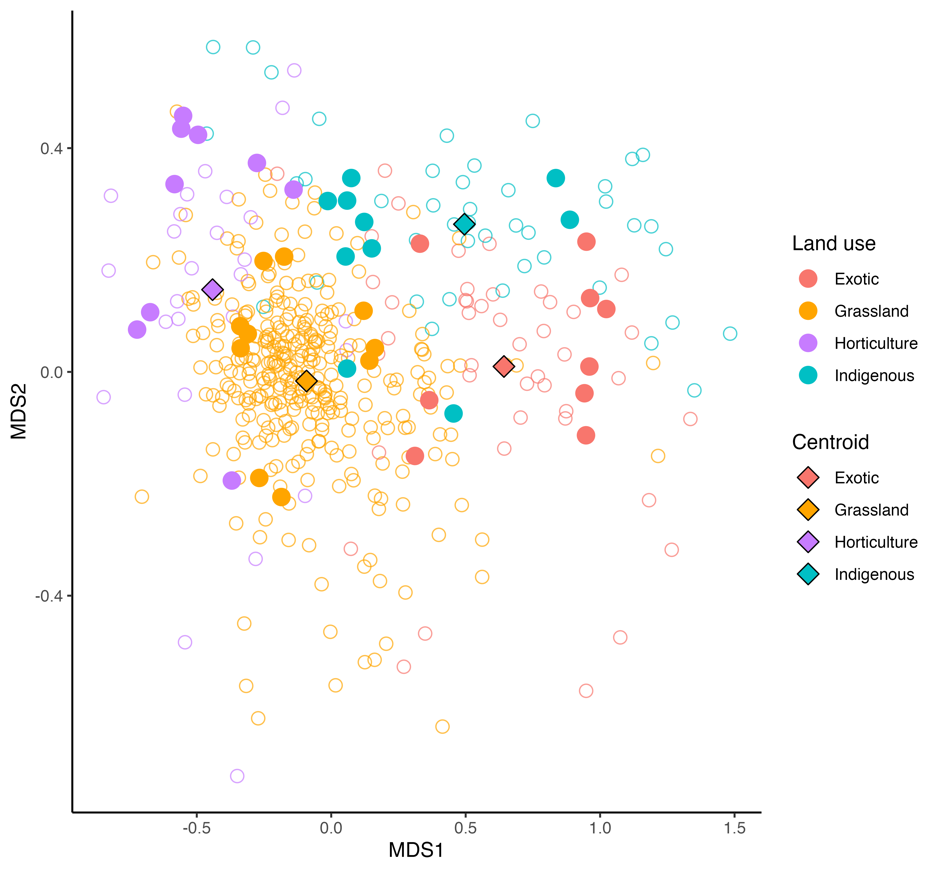


**Figure S1:** Bray-Curtis dissimilarity based non-metric multidimensional scaling (nMDS) ordination of bacterial community composition (derived from the analysis of 16S rRNA gene fragments) from a previous large-scale project (Hermans *et al.*, 2020b). Solid-circles represent sites that were randomly subsampled from those that fell within two standard deviations of the data centroid (represented by the solid diamonds) for each land use.


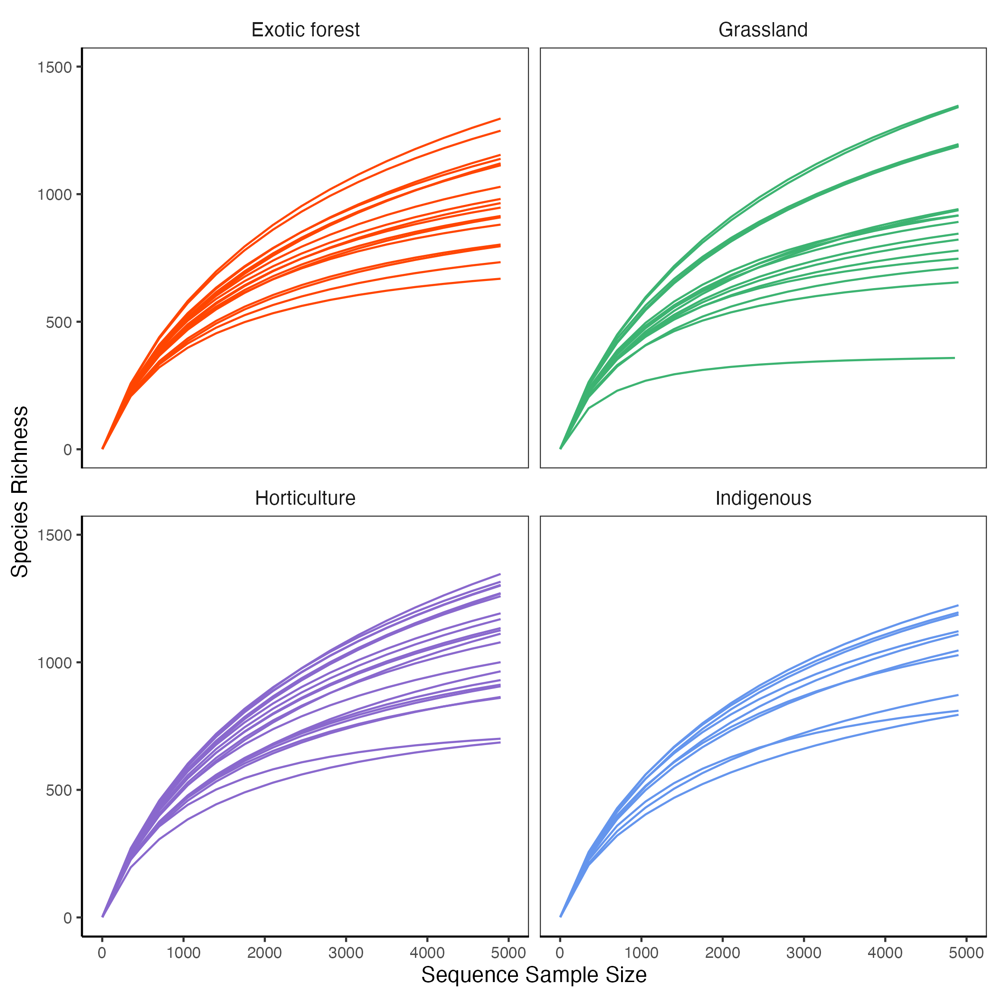


**Figure S2:** Rarefaction curves of the ASV data, displaying the relative taxon richness of bacterial communities from the analysis of up to 4875 16S rRNA gene sequences, grouped by the current land use of the sites, whether long-term or converted.


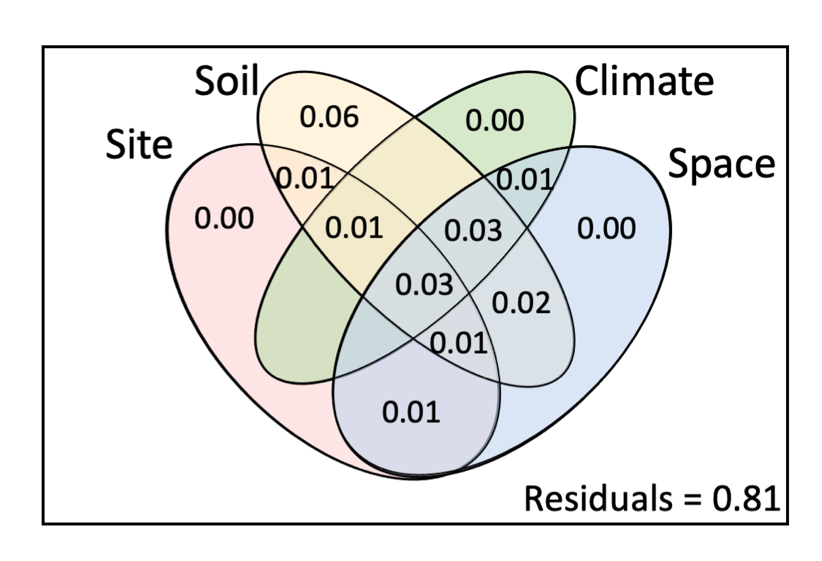


**Figure S3:** The amount of variation in bacterial community composition, based on variance partitioning (VarPart) that could be explained by site (elevation (m), aspect, slope), soil chemistry (pH, total nitrogen (%), Olsen P (mg/kg), macroporosity (% v/v), arsenic (mg/kg), cadmium (mg/kg), chromium (mg/kg), copper (mg/kg), zinc (mg/kg)), climate (Jan max temperature, June max temperature, annual average global solar radiation (MJ/m^2^/day), total rain days, total rain fall (mm) or space (Easting and Northing (NZTM)).


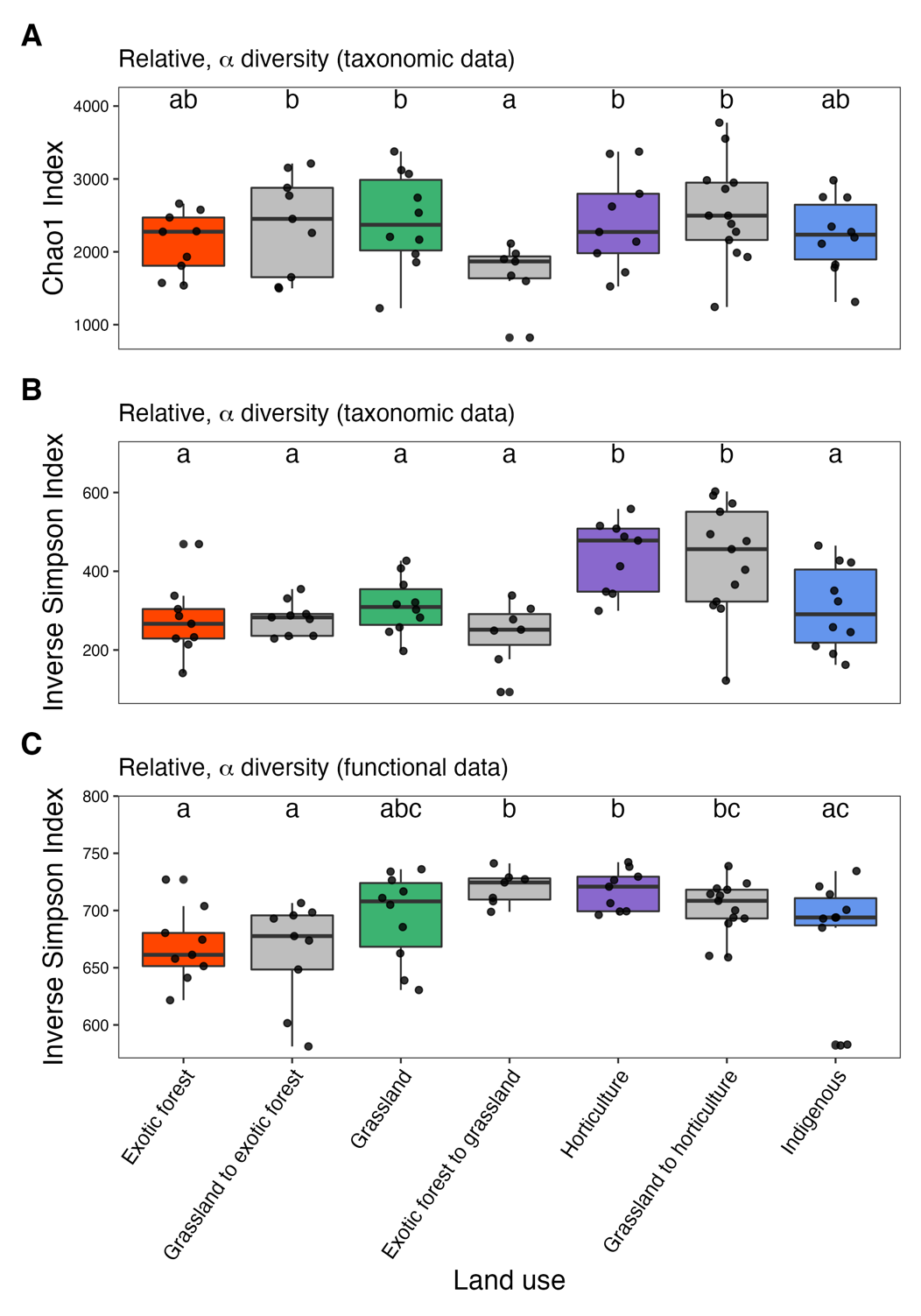


**Figure S4:** Boxplots display the estimated relative alpha diversity of the bacterial communities and functional profiles of the seven site types. The boxes represent the interquartile range (IQR: 25-75% of the data), the horizontal line indicates the median, while the whiskers extend to 1.5 times the IQR. The points represent individual values for each of the sites. (A) Chao1 index estimated ASV richness and (B) Inverse Simpson index estimated ASV evenness and (C) SEED subsystem level 4 evenness. Boxes with different letters within each panel indicate significant differences from each other (Dunn’s *P* < 0.05)

**
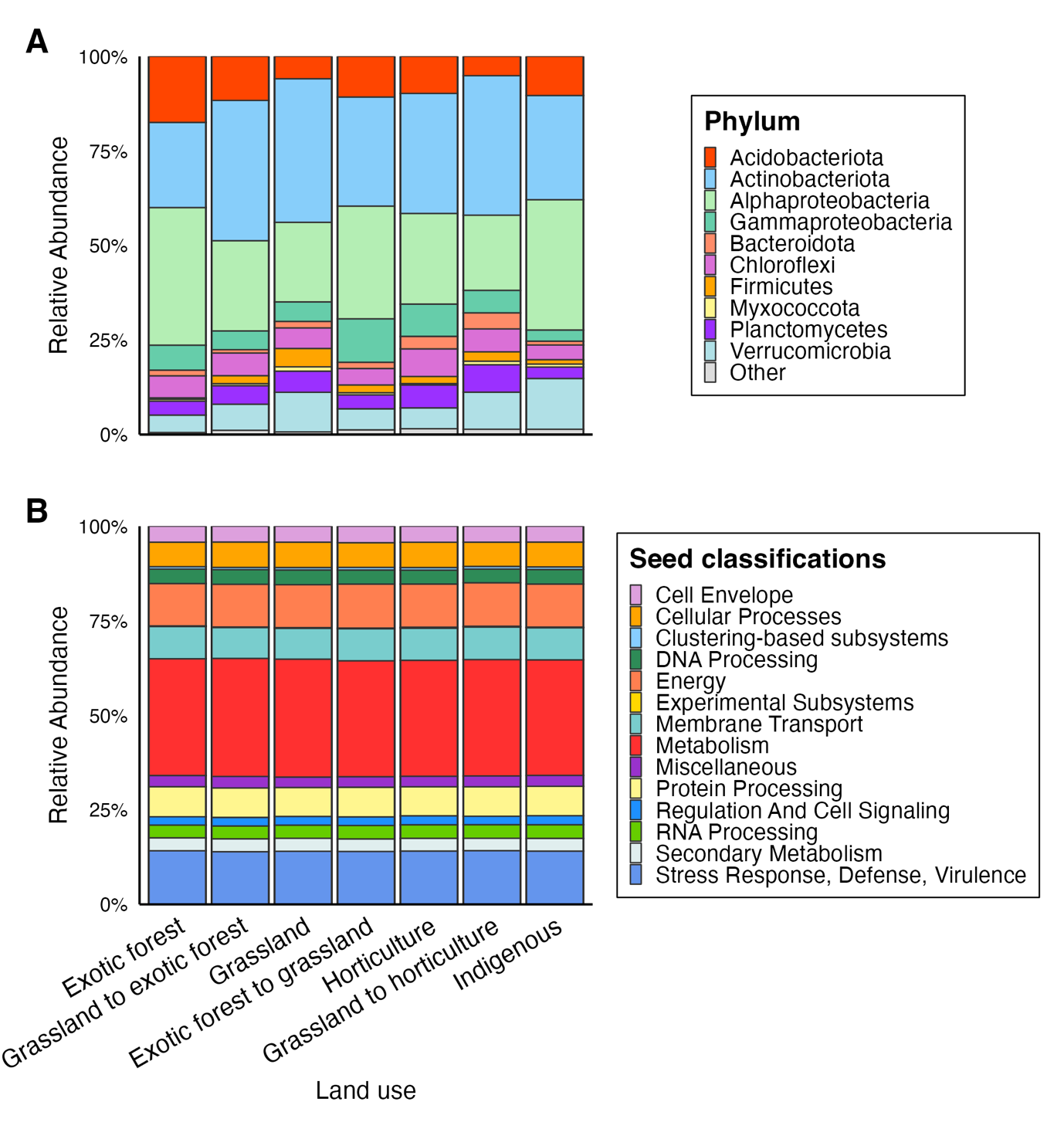
**

**Figure S5:** Stacked bar plots of (A) the relative abundance of the abundant phyla (representing >1% of the total relative abundance), with bacteria grouped by phyla, except Proteobacteria, which are split by their respective classes and (B) the relative abundance of all SEED subsystem level 1 functional categories with reads assigned.


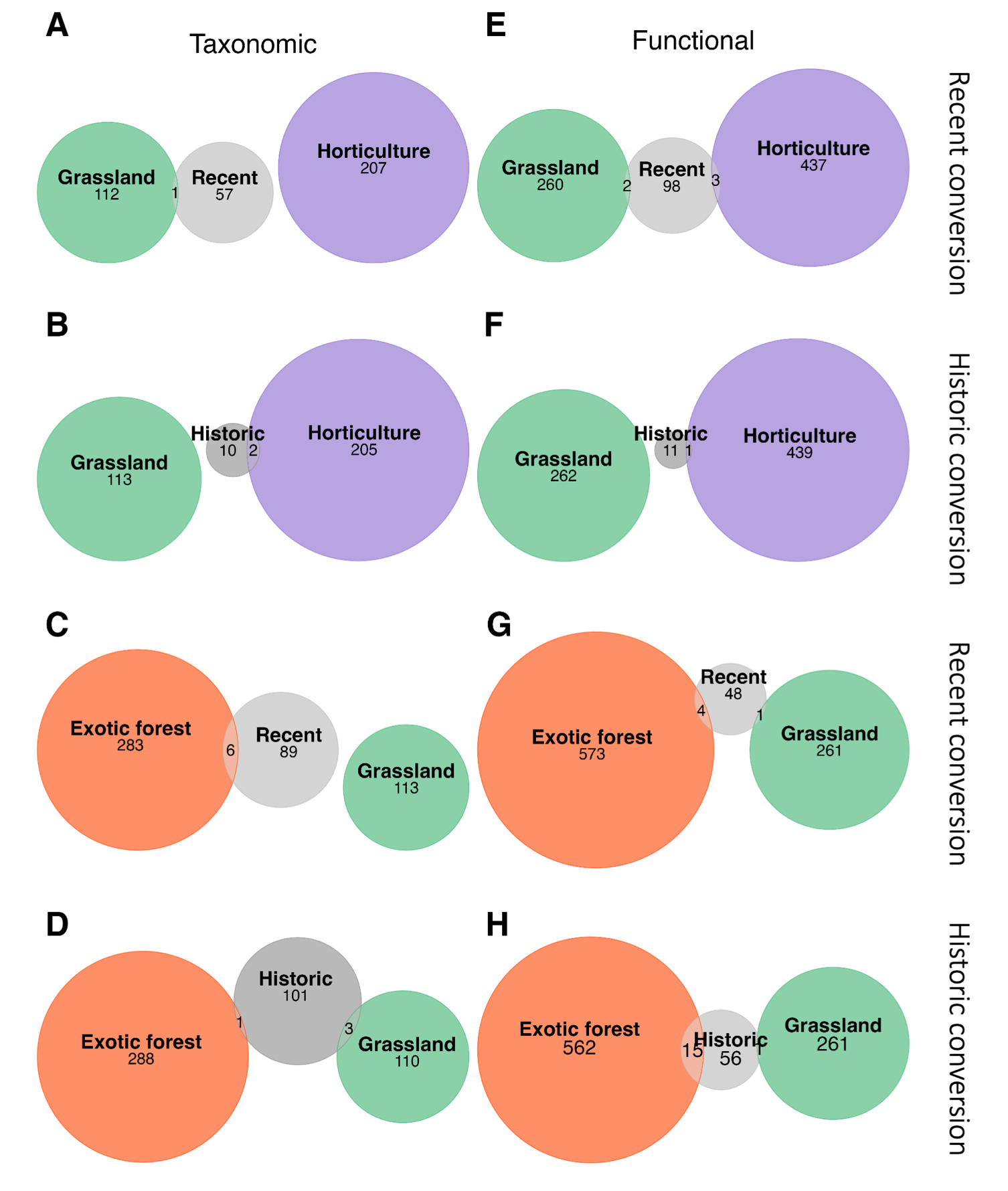


**Figure S6:** Euler diagrams indicating the observed number of shared (A-D) indicator ASVs and (E-H) indicator functional groups (based on Level 4 KEGG categories) between the recently and historically converted (A,B,E & F) grassland to horticulture sites and the (C,D,G & H) exotic forest to grassland sites and their respective long-term former and current land use sites.

**
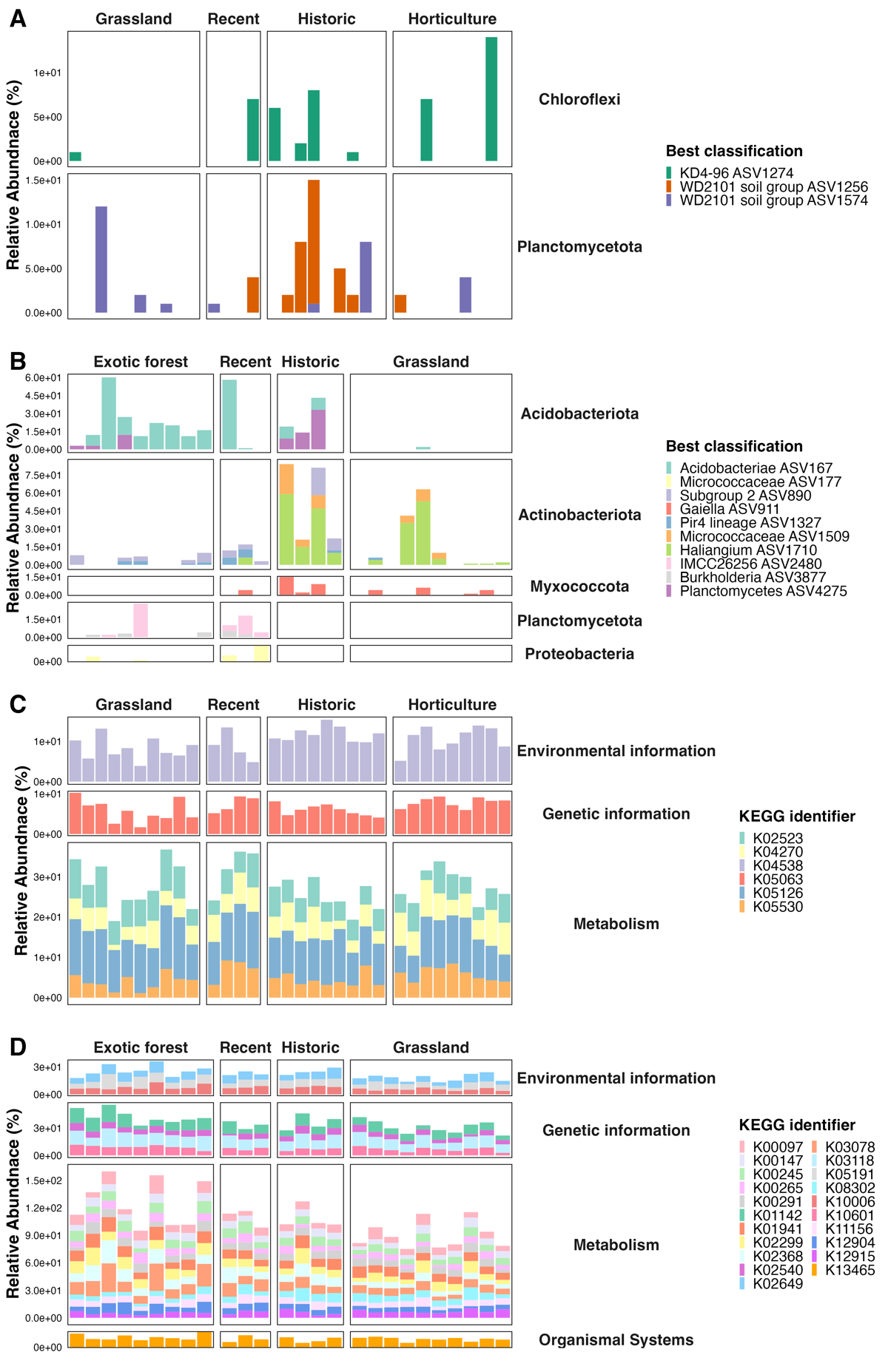
**

**Figure S7:** Stacked bar charts visualizing the mean relative abundance of the shared indicator ASVs (A & B), split by the different phyla the taxa are assigned to and the indicator functional groups (C & D), split by the Level 1 KEGG categories, between the (A & C) grassland to horticulture sites and the (B & D) exotic forest to grassland sites and each of their respective long-term sites. The converted sites are split by whether they are classified as recent or historical conversions.

References

Hermans, S.M., Buckley, H.L., Case, B.S., Curran-Cournane, F., Taylor, M., and Lear, G. (2020) Using soil bacterial communities to predict physico-chemical variables and soil quality. *Microbiome* **8**: 1–13.

Wratt, D.S., Tait, A., Griffiths, G., Espie, P., Jessen, M., Keys, J., et al. (2006) Climate for crops: integrating climate data with information about soils and crop requirements to reduce risks in agricultural decision-making. *Meteorol Appl* **13**: 305–315.
