# Supplementary material for: Land use modification causes slow, but predictable, change in soil microbial community composition and functional potential
Source: Environ Microbiome. 2023 Apr 6;18:30. doi: 10.1186/s40793-023-00485-x (PMC10080853; doi:10.1186/s40793-023-00485-x)
Supplement: Supplementary file 2 — Additional file 2. R code associated with functional data processing. [file 40793_2023_485_MOESM2_ESM.pdf]

NS_metagenomic_analyses

2022-11-29

Processing SEED funtaional/tax/sample tables

```
#Read in functional table from Megan6
otu.SEED.3<- read.delim("seed.non.normalised.240921.txt", sep = "\t", header = TRUE)
# check the structure of the object
str(otu.SEED.3)
#OTU ID's have to be the rownames
rownames(otu.SEED.3)<- otu.SEED.3$SampleID
####Function table####
functions.SEED.3 <- otu.SEED.3 %>% dplyr::select(SampleID) %>%
  separate(SampleID, c("Level1","Level2","Level3","Level4"),";")
#change to factor
functions.SEED.3<- functions.SEED.3 %>%
  mutate_if(is.character, as.factor)
str(functions.SEED.3)
#add the first column from the OTU table so that OTU and taxa tables match
functions.SEED.3<- cbind(otu.SEED.3$SampleID, functions.SEED.3)
#rename the first column
colnames(functions.SEED.3)[1]<- "SampleID"
str(functions.SEED.3)
#OTU ID's have to be the rownames, same as in the OTU table
rownames(functions.SEED.3)<- functions.SEED.3$SampleID
#delete the first columns of both OTU and functions.SEED tables
otu.SEED.3<- otu.SEED.3 %>%
  dplyr::select(-SampleID)
functions.SEED.3<- functions.SEED.3 %>%
  dplyr::select(-SampleID)
####Metadata table####
meta_chem <- read.table("../metagenomics_mapping_chem.txt", sep='\t',
                        header=TRUE, row.names = 1, stringsAsFactors = TRUE)
meta_chem = meta_chem[order(row.names(meta_chem)),]##Sort by site alphabetically
```

Handoff to phyloseq

```
otu_mat.SEED.3<- as.matrix(otu.SEED.3)
fun_mat.SEED.3<- as.matrix(functions.SEED.3)
#transform data to phyloseq objects
phylo_OTU.SEED.3<- otu_table(otu_mat.SEED.3, taxa_are_rows = TRUE)
sample_names(phylo_OTU.SEED.3)<- row.names(meta_chem)
phylo_FUN.SEED.3<- tax_table(fun_mat.SEED.3)
phylo_samples.SEED.3<- sample_data(meta_chem)
#and put them in one object
ps.SEED.3 <- phyloseq(otu_table(otu_mat.SEED.3, taxa_are_rows=TRUE),
                      sample_data(meta_chem), tax_table(fun_mat.SEED.3))
```

```
head(sample_data(ps.SEED.3))
#check if everything looks good
sample_sums(ps.SEED.3)       #should sum up the number of all reads per sample
sample_names(ps.SEED.3)      #sample names
rank_names(ps.SEED.3)        #taxa levels
sample_variables(ps.SEED.3)  #factors

ps.SEED = ps.SEED.3
##Relative abundance
ps.RA  = transform_sample_counts(ps.SEED.3, function(x) x / sum(x) )
```

CSS normalisation

```
library(metagenomeSeq)
#remove singletons
pruned.all<-filter_taxa(ps.SEED, function(x) sum(x) > 2, TRUE)
MGS <- phyloseq_to_metagenomeSeq(ps.SEED)

p <- cumNormStatFast(MGS)
MGS.norm.all <- cumNorm(MGS, p =p)
norm.all <- MRcounts(MGS.norm.all, norm = TRUE, log = FALSE)
data_norm.all <- pruned.all
otu_table(data_norm.all) <- otu_table(norm.all, taxa_are_rows = T)

# CSS will sometimes decrease the fold-difference in sampling depth but not always.
max(sample_sums(ps.SEED))/min(sample_sums(ps.SEED))
max(sample_sums(pruned.all))/min(sample_sums(pruned.all))
max(sample_sums(data_norm.all))/min(sample_sums(data_norm.all))

css.SEED <-transform_sample_counts(data_norm.all, function(x) x / sum(x))
```

Alpha diversity

```
richness = estimate_richness(ps.RA, measures = c( "InvSimpson"))
met.2 = as.data.frame(sample_data(ps.RA))
met.richness <- as.data.frame(cbind(richness,met.2))

#Dunn test Inverse Simpson
alpha.dunn = dunnTest(InvSimpson ~ Clean_names,
                      data=met.richness,method="holm")
alpha.dunn2= alpha.dunn$res
alpha.letters = cldList(P.unadj ~ Comparison,
                        data=alpha.dunn2,threshold  = 0.05)
alpha.letters$Clean_names = c("Exotic","Exotic to Grassland", "Grassland",
                              "Grassland to Exotic","Grassland to Horticulture",
                              "Horticulture","Indigenous")
#Add landuse for x axis placement
alpha.letters$Site_type = c("Representative","Transitional","Representative",
                            "Transitional","Transitional","Representative",
                            "Representative")
#Add colours in order of levels
levels(met.richness$Clean_names)
colours.alpha = c("orangered1","grey","mediumseagreen","grey",
```

```
                   "mediumpurple3","grey","cornflowerblue")

ggplot(met.richness, aes(x = Clean_names, y = InvSimpson,  fill= Clean_names)) +
  geom_boxplot(aes(fill=Clean_names)) + theme_bw()+ labs(x = "Land use")+
  theme(panel.grid.major = element_blank(), panel.grid.minor = element_blank(),
       legend.position = "none", axis.text.x = element_text(angle = 55, hjust=1, size = 11),
       axis.text= element_text(colour="black"),
       axis.title=element_text(size=15, colour = "black"))+ geom_jitter(width = 0.2,  alpha =0.8)+
  theme(strip.background = element_blank(),strip.text.x = element_text(size=12))+
  labs(y = "Inverse Simpson Index", fill = "Land use", x= "Land use") +
  ggtitle(expression(paste( "Relative, ", alpha, " diversity (functional data)"))) +
  scale_x_discrete(labels=c("Exotic forest", "Grassland to exotic forest","Grassland",
                            "Exotic forest to grassland", "Horticulture",
                            "Grassland to horticulture", "Indigenous"))+
  geom_text(data=alpha.letters, aes(label = Letter, x = Clean_names, y = 790), size = 5) +
  scale_fill_manual(values = colours.alpha)
```

NMDS with environmental vectors

```
otu = as(otu_table(css.SEED),"matrix")
# transpose if necessary
if(taxa_are_rows(css.SEED)){otu <- t(otu)}
# Coerce to data.frame
otu.df = as.data.frame(otu)

MDS<-metaMDS(otu.df, distance="bray", k=2, trymax=999, autotransform = FALSE)
MDS_points<-data.frame(MDS$points)
MDS_map<-cbind(MDS_points,meta.67)

otu.df = otu.df[order(row.names(otu.df)),]
chem.stand<-decostand(meta.67[,13:24], margin=2, method="standardize", na.rm=T)
chem.stand = chem.stand[order(row.names(chem.stand)),]
identical(row.names(otu.df),row.names(chem.stand))
names(chem.stand)<-c("pH", "TN", "OlsenP*", "CtoN", "NO3-N", "NH4-N*",
                     "Macroporosity", "Arsenic*","Cadmium*", "Chromium*",
                     "Copper*", "Zinc"  )
fit <- envfit(MDS, chem.stand,perm = 999, na.rm=T)
scores(fit, "vectors")
env.scrs <- as.data.frame(scores(fit, display = "vectors"))
env.scrs <- cbind(env.scrs, Variable = rownames(env.scrs))

#only significant pvalues
#shortcutting ef$vectors
A <- as.list(fit$vectors)
#creating the dataframe
pvals<-as.data.frame(A$pvals)
arrows<-as.data.frame(A$arrows*sqrt(A$r))
C<-cbind(arrows, pvals)
#subset
Cred<-subset(C,pvals<0.05)
rownames(Cred)[6] = "Macroporosity"
rownames(Cred)[2] = "Total nitrogen"
Cred <- cbind(Cred, Variable = rownames(Cred))
```

```r
nmds.labels = c("Exotic forest","Grassland to exotic forest",
                "Grassland","Exotic forest to grassland","Horticulture",
                "Grassland to horticulture","Indigenous")

levels(MDS_map$Clean_names)
MDS_map$Clean_names <- factor(MDS_map$Clean_names,
                              levels = c("Exotic","Grassland to Exotic","Grassland",
                                         "Exotic to Grassland","Horticulture",
                                         "Grassland to Horticulture","Indigenous"))
nmds.seed = ggplot(MDS_map, aes(MDS1, MDS2)) +
  geom_point(aes(shape = Clean_names, color = Clean_names), size=2) +
  scale_color_manual(labels = nmds.labels, values=(values=colours.merged)) +
  labs(fill="Land use") +
  scale_shape_manual(labels = nmds.labels,values = c(16,24,16,24,16,24,16,24))+
  geom_segment(data = Cred, aes(x = 0, xend = NMDS1/2, y = 0, yend = NMDS2/2),
             arrow = arrow(length = unit(0.25, "cm")), colour = "gray", size=0.5) +
  annotate("text", x=0.28, y=0.22, label= "P = 0.001") +
  annotate("text", x=0.26, y=0.25, label= "stress = 0.18") +
  geom_text_repel(data = Cred, aes(x = NMDS1/2, y = NMDS2/2, label = Variable),
                max.overlaps = Inf, alpha = 0.8, size = 4, color = "black") +
  theme_bw() +  labs(y = "NMDS2", x= "NMDS1",colour = "Land use", shape = "Land use",
                    subtitle = "Functional data")
```

Subsetting for each transition and their long-term counterparts

```r
ps.GE.longterm = subset_samples(css.SEED, Transition == "G->E" |
                                  Transition == "Grassland" |
                                  Transition == "Exotic")
ps.GH.longterm = subset_samples(css.SEED, Transition == "G->H" |
                                  Transition == "Grassland" |
                                  Transition == "Horticulture")
ps.EG.longterm = subset_samples(css.SEED, Transition == "E->G" |
                                  Transition == "Exotic" |
                                  Transition == "Grassland")
```

Single axis plots

```r
library(FSA)
library(rcompanion)
#Single axis NMDS points Grassland to Exotic
MDS.GE = ordinate(ps.GE.longterm, method = "NMDS", distance = "bray",
                trymax = 999, autotransform = F)
MDS.points.EG=data.frame(MDS.GE$points)
met.EG = as.data.frame(sample_data(ps.GE.longterm))
MDS.GE.df<-cbind(MDS.points.EG,met.EG)
MDS.GE.df[,'Transition.age']<-factor(MDS.GE.df[,'Transition.age'])
MDS.GE.df =MDS.GE.df %>%
  mutate(Transition.age = coalesce(Transition.age, Transition))

#Dunn's test EG
pt = dunnTest(MDS1 ~ Transition.age, data=MDS.GE.df, method = "holm")
PT3= pt$res
```

```
GE.mds.letters = cldList(P.adj ~ Comparison, data=PT3,threshold  = 0.05)
#Significance letters - manually do letters so they are in alphabetical order on the plot
GE.letter.names = c("Grassland","Recent", "Historic", "Exotic")
Letter <- c("a", "a","ab","b")
GE.single.letter = data.frame(GE.letter.names, Letter)

levels(MDS.GE.df$Transition.age)
MDS.GE.df$Transition.age <- factor(MDS.GE.df$Transition.age,
                                   levels = c("Grassland","Recent","Historic","Exotic"))
ggplot(data = MDS.GE.df, aes(Transition.age,MDS1)) +
  geom_point(aes(colour = Transition.age ), size=2) +
  scale_color_manual(values=c("mediumseagreen","dimgrey","darkgrey","orangered1"))+
  theme_bw() + labs(colour = "Land Use")+ theme(axis.title.x=element_blank()) +
  geom_text(data=GE.single.letter, aes(label = Letter, x = Group, y = 0.2), size = 5) +
  labs(y = "NMDS1")

#Single axis NMDS points Grassland to Horticulture
MDS.GH = ordinate(ps.GH.longterm, method = "NMDS", distance = "bray",
                  trymax = 999, autotransform = F)
MDS.points.GH=data.frame(MDS.GH$points)
met.GH = as.data.frame(sample_data(ps.GH.longterm))
MDS.GH.df<-cbind(MDS.points.GH,met.GH)
MDS.GH.df[,'Transition.age']<-factor(MDS.GH.df[,'Transition.age'])
MDS.GH.df =MDS.GH.df %>%
  mutate(Transition.age = coalesce(Transition.age, Transition))

#Dunn's test EG
pt = dunnTest(MDS1 ~ Transition.age, data=MDS.GH.df, method = "holm")
PT3= pt$res
Group <- c("Grassland","Recent","Historic","Horticulture")
Letter <- c("a", "a","a","a")
GH.mds.letters = data.frame(Group,Letter)
levels(MDS.GH.df$Transition.age)
MDS.GH.df$Transition.age <- factor(MDS.GH.df$Transition.age,
                                   levels = c("Grassland","Recent",
                                              "Historic","Horticulture"))

ggplot(data = MDS.GH.df, aes(Transition.age,MDS1)) +
  geom_point(aes(colour = Transition.age ), size=2) +
  scale_color_manual(values=c("mediumseagreen","dimgrey","darkgrey","mediumpurple3"))+
  theme_bw() + labs(colour = "Land Use")+ theme(axis.title.x=element_blank()) +
  geom_text(data=GH.mds.letters, aes(label = Letter, x = Group, y = 0.15), size = 5) +
  labs(y = "NMDS1")

#Single axis NMDS points Exotic to Grassland
MDS.EG = ordinate(ps.EG.longterm, method = "NMDS", distance = "bray",
                  trymax = 999, autotransform = F)
MDS.points.EG=data.frame(MDS.EG$points)
met.EG = as.data.frame(sample_data(ps.EG.longterm))
MDS.EG.df<-cbind(MDS.points.EG,met.EG)
MDS.EG.df[,'Transition.age']<-factor(MDS.EG.df[,'Transition.age'])
MDS.EG.df =MDS.EG.df %>%
  mutate(Transition.age = coalesce(Transition.age, Transition))
```

```
#Dunn's test EG
pt = dunnTest(MDS1 ~ Transition.age, data=MDS.EG.df, method = "holm")
PT3= pt$res
EG.mds.letters = cldList(P.adj ~ Comparison, data=PT3,threshold  = 0.05)

levels(MDS.EG.df$Transition.age)
MDS.EG.df$Transition.age <- factor(MDS.EG.df$Transition.age,
                                   levels = c("Exotic","Recent","Historic","Grassland"))
ggplot(data = MDS.EG.df, aes(Transition.age,MDS1)) +
  geom_point(aes(colour = Transition.age ), size=2) +
  scale_color_manual(values=c("orangered1","dimgrey","darkgrey","mediumseagreen"))+
  theme_bw() + labs(colour = "Land Use")+ theme(axis.title.x=element_blank()) +
  geom_text(data=EG.mds.letters, aes(label = Letter, x = Group, y = 0.2), size = 5) +
  labs(y = "NMDS1")
```

Process KEGG functional tables

```
otu.KEGG.3<- read.delim("kegg.otu.250921.txt", sep = "\t", header = TRUE)
str(otu.KEGG.3)
#OTU ID's have to be the rownames
rownames(otu.KEGG.3)<- otu.KEGG.3$SampleID
####Function table####
functions.KEGG.3 <- otu.KEGG.3 %>% dplyr::select(SampleID) %>%
  separate(SampleID, c("Level1","Level2","Level3","Level4"),";")
functions.KEGG.3<- functions.KEGG.3 %>%
  mutate_if(is.character, as.factor)
str(functions.KEGG.3)
#add the first column from the OTU table so that OTU and taxa tables match
functions.KEGG.3<- cbind(otu.KEGG.3$SampleID, functions.KEGG.3)
#rename the first column
colnames(functions.KEGG.3)[1]<- "SampleID"
str(functions.KEGG.3)
#OTU ID's have to be the rownames, same as in the OTU table
rownames(functions.KEGG.3)<- functions.KEGG.3$SampleID
otu.KEGG.3<- otu.KEGG.3 %>%
  dplyr::select(-SampleID)
functions.KEGG.3<- functions.KEGG.3 %>%
  dplyr::select(-SampleID)

####Phyloseq KEGG 3 levels####
otu_mat.KEGG.3<- as.matrix(otu.KEGG.3)
fun_mat.KEGG.3<- as.matrix(functions.KEGG.3)
#transform data to phyloseq objects
phylo_OTU.KEGG.3<- otu_table(otu_mat.KEGG.3, taxa_are_rows = TRUE)
sample_names(phylo_OTU.KEGG.3)<- row.names(meta_chem)
phylo_FUN.KEGG.3<- tax_table(fun_mat.KEGG.3)
phylo_samples.KEGG.3<- sample_data(meta_chem)
#and put them in one object
ps.KEGG.3 <- phyloseq(otu_table(otu_mat.KEGG.3, taxa_are_rows=TRUE),
                      sample_data(meta_chem), tax_table(fun_mat.KEGG.3))
head(sample_data(ps.KEGG.3))
#check if everything looks good
sample_sums(ps.KEGG.3)       #should sum up the number of all reads per sample
```

```r
sample_names(ps.KEGG.3)      #sample names
rank_names(ps.KEGG.3)        #taxa levels
sample_variables(ps.KEGG.3)  #factors
##Relative abundance
ps.kegg.RA  = transform_sample_counts(ps.KEGG, function(x) x / sum(x) )
kegg.RA.names = ps.kegg.RA
kegg.names <-taxa_names(kegg.RA.names)
taxa_names(kegg.RA.names) <- paste0("KEGG", seq(ntaxa(kegg.RA.names)))

#CSS normalisation
MGS <- phyloseq_to_metagenomeSeq(ps.KEGG.3)
p <- cumNormStatFast(MGS)
MGS.norm.all <- cumNorm(MGS, p =p)
norm.all <- MRcounts(MGS.norm.all, norm = TRUE, log = FALSE)
data_norm.all <- pruned.all
otu_table(data_norm.all) <- otu_table(norm.all, taxa_are_rows = T)
css.kegg = data_norm.all

max(sample_sums(ps.KEGG))/min(sample_sums(ps.KEGG))
max(sample_sums(pruned.all))/min(sample_sums(pruned.all))
max(sample_sums(data_norm.all))/min(sample_sums(data_norm.all))
css.kegg.RA <-transform_sample_counts(css.kegg, function(x) x / sum(x) )
```

Indicator funcations KEGG

```r
ps.KEGG.rep.names = subset_samples(css.kegg.RA, Site_type == "Representative")
ps.KEGG.tran.names = subset_samples(css.kegg.RA, Site_type == "Transitional")

library(plyr)
library(indicspecies)
#Extract OTU tables from each ps object
OTU1 = as(otu_table(ps.KEGG.tran.names), "matrix")
# transpose if necessary
if(taxa_are_rows(ps.KEGG.tran.names)){OTU1 <- t(OTU1)}
# Coerce to data.frame
rep.otu.KEGG.df = as.data.frame(OTU1)
tran.otu.KEGG.df = as.data.frame(OTU1)
#Remove zeros
rep.otu.KEGG.df = rep.otu.KEGG.df[, !apply(rep.otu.KEGG.df==0,2,all)]
tran.otu.KEGG.df = tran.otu.KEGG.df[, !apply(tran.otu.KEGG.df==0,2,all)]
#Get mapping files
rep.met.KEGG = as(sample_data(ps.KEGG.rep.names), "data.frame")
tran.met.KEGG = as(sample_data(ps.KEGG.tran.names), "data.frame")
identical(row.names(tran.met.KEGG), rownames(tran.otu.KEGG.df))
#Extract land uses for each and assign a numerical value
landuse.rep = rep.met.KEGG$Transition
landuse.tran = tran.met.KEGG$Transition
#Run indval analysis for multiple groups
(iva.rep.KEGG <- multipatt(rep.otu.KEGG.df,landuse.rep, max.order = 1,
                           control = how(nperm = 999)))
(iva.tran.KEGG <- multipatt(tran.otu.KEGG.df,landuse.tran, max.order = 1 ,
                            control= how(nperm = 999)))
# Export the result to file
```

```
summary(iva.rep.KEGG, indvalcomp = TRUE)
options(max.print = 9999)
capture.output(summary(iva.rep.KEGG, indvalcomp = TRUE),
               file="indicsummary_representative_KEGG")
capture.output(summary(iva.tran.KEGG, indvalcomp = TRUE),
               file="indicsummary_transitional_KEGG")

#Repeat for sites split by transition history
history.landuse.tran = tran.met.kegg$Transition_history
(iva.hist.KEGG <- multipatt(tran.otu.KEGG.df,history.landuse.tran,max.order = 1,
                            control = how(nperm = 999)))
capture.output(summary(iva.hist.KEGG, indvalcomp = TRUE),
               file="indicsummary_history_KEGG")



#Subsetting data
ind.combined.seed <- read.table("indicator.combined.KEGG.max1.txt", sep='\t',
                                header=TRUE,  stringsAsFactors = TRUE)
library(eulerr)
set.seed(1)
ind.combined.kegg

exotic.venn.kegg = subset(ind.combined.seed, Landuse == "Exotic")
grass.venn.kegg = subset(ind.combined.seed, Landuse == "Grassland")
hort.venn.kegg = subset(ind.combined.kegg, Landuse == "Horticulture")
#Subset by historic split
EG.h.kegg= subset(ind.combined.seed,Landuse == "Historic_E->G")
EG.r.kegg=subset(ind.combined.seed,Landuse == "Recent_E->G")
GE.h.kegg= subset(ind.combined.kegg,Landuse == "Historic_G->E")
GE.r.kegg=subset(ind.combined.kegg,Landuse == "Recent_G->E")
GH.h.kegg= subset(ind.combined.kegg,Landuse == "Historic_G->H")
GH.r.kegg=subset(ind.combined.kegg,Landuse == "Recent_G->H")
```

Venn diagram of indicator functions

```
#Exotic to grassland
#Colours + labels
cols.2 <- c(Exotic = "orangered1", Grassland = "mediumseagreen",
            EG.h.v = "gray54",EG.r.v = "gray72")
labs.2 <- c(Exotic = "Exotic", Grassland = "Grassland",
            EG.h.v = "Historic conversion",EG.r.v = "Recent conversion")
labs.eg.2 <- c(Exotic = "Exotic", Grassland = "Grassland",
               EG.h.v = "Historic conversion")
labs.eg.3 <- c(Exotic = "Exotic", Grassland = "Grassland",
               EG.r.v = "Recent conversion")
#Historic
cols.3.kegg <- c(Exotic = "orangered1", EG.h.v = "gray72", Grassland = "mediumseagreen")
labs.3.kegg <- c(Exotic = "Exotic forest",EG.h.v = "Historic", Grassland = "Grassland")
eg.2.kegg<- list(Exotic=exotic.venn.kegg$Function,
          EG.h.v=EG.h.kegg$Function,
          Grassland=grass.venn.kegg$Function)
venn.eg.historic.kegg = plot(euler(eg.2.kegg), quantities = list(cex = 1.5),
```

```r
     shape = "ellipse",
     alpha = 0.6, fills = cols.3.kegg,labels = list(labels = labs.3.kegg,fontsize =18 ),
     edges = cols.3.kegg)
plot(euler(eg.2.kegg), quantities = list(cex = 1.5),
     shape = "ellipse",
     alpha = 0.6, fills = cols.3.kegg,
     edges = cols.3.kegg)
#Recent
cols.3 <- c(Exotic = "orangered1", Grassland = "mediumseagreen", EG.r.v = "gray72")
labs.3 <- c(Exotic = "Exotic forest", Grassland = "Grassland",EG.r.v = "Recent")
eg.3.kegg<- list(Exotic=exotic.venn.kegg$Function,
           Grassland=grass.venn.kegg$Function,
           EG.r.v=EG.r.kegg$Function)
venn.eg.recent.kegg = plot(euler(eg.3.kegg), quantities = list(cex = 1.2),
     shape = "ellipse",
     alpha = 0.6, fills = cols.3,labels = list(labels = labs.3,fontsize =18),
     edges = cols.3)

#Grassland to exotic
#Colours + labels
cols.4 <- c(Grassland = "mediumseagreen", ge.h.v = "gray54",
            ge.r.v = "gray72", Exotic = "orangered1")
labs.4 <- c(Grassland = "Grassland",  ge.h.v = "Historic conversion",
            ge.r.v = "Recent conversion",Exotic = "Exotic")
#Historic
ge.2.kegg<- list(Grassland=grass.venn.kegg$Function,
            ge.h.v=GE.h.kegg$Function,
           Exotic=exotic.venn.kegg$Function )
cols.ge.2 <- c("mediumseagreen","gray54", "orangered1" )
labs.ge.2 <- c( Grassland = "Grassland",ge.r.v = "Historic",
                Exotic = "Exotic forest")
venn.ge.historic.kegg = plot(euler(ge.2.kegg), quantities = list(cex = 1.5),
     shape = "ellipse",
     alpha = 0.6, fills = cols.ge.2,labels = list(labels = labs.ge.2,fontsize =18 ),
     edges = cols.ge.2)
#Recent
ge.3.kegg<- list(Grassland=grass.venn.kegg$Function,
            ge.r.v=GE.r.kegg$Function,
            Exotic=exotic.venn.kegg$Function )
cols.ge.3 <- c("mediumseagreen","gray72", "orangered1")
labs.ge.3 <- c("Grassland","Recent","Exotic forest")
venn.ge.recent.kegg = plot(euler(ge.3.kegg), quantities = list(cex = 1.5),
     shape = "ellipse", alpha = 0.6, fills = cols.ge.3,
     labels = list(labels = labs.ge.3,fontsize =18 ),
     edges = cols.ge.3,main = list(label = "Functional", cex = 2))

#Grassland to horticulture
#Historic
gh.2.kegg<- list(Grassland=grass.venn.kegg$Function,
           gh.h.v=GH.h.kegg$Function,
          Horticulture=hort.venn.kegg$Function)
cols.gh.2 <- c("mediumseagreen","gray54","mediumpurple3" )
labs.gh.2 <- c("Grassland","Historic","Horticulture")
```

```
venn.gh.historic.kegg = plot(euler(gh.2.kegg), quantities = list(cex = 1.2),
     shape = "ellipse",
     alpha = 0.6, fills = cols.gh.2,labels = list(labels = labs.gh.2,fontsize =18 ),
     edges = cols.gh.2)
#Recent
gh.3.kegg<- list(Grassland=grass.venn.kegg$Function,
          gh.r.V=GH.r.kegg$Function,
          Horticulture=hort.venn.kegg$Function)
cols.gh.3 <- c("mediumseagreen", "gray72","mediumpurple3" )
labs.gh.3 <- c("Grassland", "Recent", "Horticulture")
venn.gh.recent.kegg = plot(euler(gh.3.kegg), quantities = list(cex = 1.2),
     shape = "ellipse",
     alpha = 0.6, fills = cols.gh.3,labels = list(labels = labs.gh.3,fontsize =18),
     main = list(label = "Functional", cex = 2),edges = cols.gh.3)
venn.gh.recent.kegg
```

Indicator bar plots

```
 #Function for plot_bar without colour selected as black to remove black outlines around bars
my_plot_bar = function (physeq, x = "Sample", y = "Abundance", fill = NULL, title = NULL,
                      facet_grid = NULL) {
    mdf = psmelt(physeq)
    p = ggplot(mdf, aes_string(x = x, y = y, fill = fill))
    p = p + geom_bar(stat = "identity", position = "stack")
    p = p + theme(axis.text.x = element_text(angle = -90, hjust = 0))
    if (!is.null(facet_grid)) {
        p <- p + facet_grid(facet_grid)
    }
    if (!is.null(title)) {
        p <- p + ggtitle(title)
    }
    return(p)
}

#Subset by shared indicator functions
shared.kegg.names <- read.table("shared_KEGG_names.txt", sep='\t',
                                header=TRUE,  stringsAsFactors = TRUE)
ind.kegg = as(shared.kegg.names$Function, 'character')
#Subset file by transition
ind.GE = subset(shared.kegg.names, shared.kegg.names$Transition == "GE")
ind.GH = subset(shared.kegg.names, shared.kegg.names$Transition == "GH")
ind.EG = subset(shared.kegg.names, shared.kegg.names$Transition == "EG")
#Subset ps object by transition type
GE.merged.kegg = subset_samples(ps.KEGG, Transition == "G->E" | Transition == "Exotic" |
                                  Transition == "Grassland")
GH.merged.kegg = subset_samples(ps.KEGG, Transition == "G->H" | Transition == "Horticulture" |
                                  Transition == "Grassland")
EG.merged.kegg = subset_samples(ps.KEGG, Transition == "E->G" | Transition == "Exotic" |
                                  Transition == "Grassland")

ind.shared.kegg.GE = subset_taxa(GE.merged.kegg, Level4 %in% ind.GE$Function)
ind.shared.kegg.GH = subset_taxa(GH.merged.kegg, Level4 %in% ind.GH$Function)
ind.shared.kegg.EG = subset_taxa(EG.merged.kegg, Level4 %in% ind.EG$Function)
```

```r
library(scales)
ge.K.names = c("K02523","K04270","K04538","K05063","K05126","K05530",
               "K05654","K05699","K05768", "K05771","K05846","K05877",
               "K06620","K06627","K06633","K06654","K06779","K06797",
                "K07299","K07536","K08501","K08967","K09450","K09664",
               "K10881","K10935")
col.ge = c("lightpink","lavender","darkseagreen2", "lightgray","aquamarine3", "salmon1",
           "khaki1", "lightcyan", "lightskyblue", "lightsalmon","orchid","sandybrown",
           "lightblue1","gainsboro", "cadetblue1", "lightcoral",'palevioletred1',
           "thistle1","plum1","cornflowerblue", "mediumorchid1", "orange1",
           "mediumaquamarine","tan1",'darkgoldenrod1',  "steelblue1",'cadetblue3')

col.ge = c("lightpink","lavender","darkseagreen2", "lemonchiffon3","aquamarine3",
           "salmon1", "khaki1", "lightcyan", "lightskyblue", "lightsalmon",
           "orchid","sandybrown", "lightblue1","gainsboro", "purple1",
           "lightcoral",'palevioletred1',"thistle1","plum1","cornflowerblue",
           "mediumorchid1","mediumaquamarine","tan1",'darkgoldenrod1',
           "steelblue1",'cadetblue3')

ge.lables.bar = c("Longterm_Grassland"= "Grassland","Recent_G->E"= "Recent",
                  "Historic_G->E"="Historic","Longterm_Exotic"="Exotic forest")
ge.labels.func = c('Cellular Processes '='Cellular processes',
                   'Environmental Information Processing'= 'Environmental information',
                   'Genetic Information Processing' = 'Genetic information',
                   'Human Diseases '='Human diseases','Metabolism'='Metabolism',
                   'Organismal Systems ' = 'Organismal Systems')
levels(sample_data(ind.shared.kegg.GE)$Transition_history)
sample_data(ind.shared.kegg.GE)$Transition_history <-
  factor(sample_data(ind.shared.kegg.GE)$Transition_history,
         levels = c("Longterm_Grassland","Recent_G->E",
                    "Historic_G->E","Longterm_Exotic"))
GE.ind.p.k = my_plot_bar(ind.shared.kegg.GE, fill="Level4") +
  facet_grid(Level1~Transition_history, scales = "free",space = "free",
             labeller = labeller(Level1= ge.labels.func, Transition_history = ge.lables.bar))+
        scale_y_continuous(labels = scientific, breaks = seq(0,1.0e+02, by = 2.0e+01 ))+
  scale_fill_manual(values=col.ge, labels = ge.K.names)+
        labs(y = "Relative Abundnace (%)",x =" ", size = 12, fill = "KEGG identifier")

eg.K.names = c('K00097','K00147','K00245','K00265','K00291','K01142',
               'K01941','K02299','K02368','K02540','K02649','K03078',
               'K03118','K05191','K08302','K10006','K10601','K11156',
               'K12904','K12915','K13465')
col.eg = c("lightpink","lavender","darkseagreen2", "plum1","lightgray",
           "aquamarine3", "salmon1", "khaki1", "lightcyan", "orchid",
           "lightskyblue", "lightsalmon", "lightblue1","gainsboro", "cadetblue1",
           "lightcoral",'palevioletred1',"thistle1","cornflowerblue",
           "mediumorchid1", "orange1")

eg.lables.bar = c("Longterm_Exotic"="Exotic forest","Recent_E->G"= "Recent",
                  "Historic_E->G"="Historic","Longterm_Grassland"= "Grassland")
eg.labels.func = c('Environmental Information Processing'= 'Environmental information',
                   'Genetic Information Processing' = 'Genetic information',
```

```
                    'Metabolism'='Metabolism' ,'Organismal Systems ' = 'Organismal Systems')
levels(sample_data(ind.shared.kegg.EG)$Transition_history)
sample_data(ind.shared.kegg.EG)$Transition_history <-
  factor(sample_data(ind.shared.kegg.EG)$Transition_history,
         levels = c("Longterm_Exotic","Recent_E->G",
                    "Historic_E->G","Longterm_Grassland"))
EG.ind.p.k = my_plot_bar(ind.shared.kegg.EG, fill="Level4") +
  facet_grid(Level1~Transition_history, scales = "free",space = "free",
             labeller = labeller(Level1= eg.labels.func, Transition_history = eg.lables.bar))+
         scale_y_continuous(labels = scientific, breaks = seq(0,1.5e+02, by = 3e+01 ))+
  scale_fill_manual(values=col.eg, labels = eg.K.names)+
        labs(y = "Relative Abundnace (%)",x =" ", size = 12, fill = "KEGG identifier")

gh.lables.bar = c("Longterm_Grassland"="Grassland","Recent_G->H"= "Recent",
                  "Historic_G->H"="Historic","Longterm_Horticulture"= "Horticulture")
gh.labels.func = c('Environmental Information Processing'= 'Environmental information',
                   'Genetic Information Processing' = 'Genetic information'
                   ,'Metabolism'='Metabolism')
levels(sample_data(ind.shared.kegg.GH)$Transition_history)
sample_data(ind.shared.kegg.GH)$Transition_history <-
  factor(sample_data(ind.shared.kegg.GH)$Transition_history,
         levels = c("Longterm_Grassland","Recent_G->H",
                    "Historic_G->H","Longterm_Horticulture"))
GH.K.names = c("K00104","K01029","K02020","K03144","K03766","K13745")

GH.ind.p.k =my_plot_bar(ind.shared.kegg.GH, fill="Level4") +
  facet_grid(Level1~Transition_history, scales = "free",space = "free",
             labeller = labeller(Level1= gh.labels.func, Transition_history = gh.lables.bar))+
  geom_bar(stat="identity")+
  scale_y_continuous(labels = scientific, breaks = seq(0,3e+01, by = 1e+01 ))+
  scale_fill_brewer(palette = "Set3", labels = ge.K.names)+
        labs(y = "Relative Abundnace (%)",x =" ", size = 12, fill = "KEGG identifier")
```

Metabolism groups significantly different

```
#Subset by metabolism
css.metabolism = subset_taxa(css.kegg, Level1=="Metabolism")
css.met.RA <-transform_sample_counts(css.metabolism, function(x) x / sum(x) )
css.met.RA.g.e = subset_samples(css.met.RA, Transition == "Grassland" |
                                  Transition == "Exotic" )
css.kegg.g.e =  subset_samples(css.kegg, Transition == "Grassland" |
                                 Transition == "Exotic" )

RANK = 'Level3'
dat <- css.met.RA.g.e %>%
  tax_glom(taxrank = RANK) %>%
  psmelt()
dat %>%
  group_by_(RANK) %>%
  do(tidy(kruskal.test(Abundance ~ Transition, data=.))) %>%
  ungroup() %>%
  mutate(p.adjust=p.adjust(p.value)) -> kw.results
#Summarize results
```

```
kw.results %>%
  subset(p.value < 0.05) %>%
  knitr::kable()
#Extract significant values
kw.ind.exotic = kw.results %>%
  subset(p.value < 0.05)

#Just include metabolism related groups
ps.metabolism = subset_taxa(css.kegg.g.e, Level1 =="Metabolism")
#just include significantly diff groups
ps.metabolism.kw <- subset_taxa(ps.metabolism,
                                Level3 %in% kw.ind.exotic$Level3)
ps.meta.kw.3 = tax_glom(ps.metabolism.kw, taxrank = "Level3")
ps.meta.kw.melt = psmelt(ps.meta.kw.3)
write.csv(ps.meta.kw.melt, "metabolism.sig.diff.leve3.csv")
metab.sig.diff.df = read.csv("metabolism.sig.diff.leve3.csv")

#Significantly different metabolism groups
plot.low = ggplot(subset(metab.sig.diff.df, Clean_groups != "Nitrogen"&
                           Clean_groups != "Methane" &
                           Clean_groups != "Porphyrin and chlorophyll"),
                  aes(x = Clean_groups, y = Abundance, fill = Clean_names)) +
   geom_boxplot(lwd=0.2, outlier.shape = NA) +theme_bw()+
  facet_grid(~Type,scales = "free_x", space = "free_x")+
  scale_fill_manual(values = c("orangered1","mediumseagreen"),
                    labels=c('Exotic forest', 'Grassland'))+
  labs(y="Relative abundance %" , x = "")
plot.high =  ggplot(subset(metab.sig.diff.df, Clean_groups == "Nitrogen" |
                             Clean_groups == "Methane"|
                             Clean_groups == "Porphyrin and chlorophyll"),
                    aes(x = Clean_groups, y = Abundance, fill = Clean_names)) +
   geom_boxplot(lwd=0.2, outlier.shape = NA) +theme_bw()+
  facet_grid(~Type,scales = "free_x", space = "free_x")+
  scale_fill_manual(values = c("orangered1","mediumseagreen"))+
  labs(y="Relative abundance %" , x = "")

#Run same code above for significantly diff nitrogen and methane groups
#Significantly different nitrogen groups plot
names.nit = c("nirA","narG/nxrA","nosZ","NR","hao")
labels.nit.cyc = c("Exotic forest", "Grassland to exotic","Exotic to grassland","Grassland")
colour.nit = c("orangered1","gray60","gray46", "mediumseagreen")
nitrogen.p = ggplot(nit.mean.sd, aes(x=mean, y=Gene, fill=LandUse)) +
  geom_bar(stat="identity", position=position_dodge()) +
  labs(x= "Relative Abundance", y= "Nitrogen cycling genes")+
  scale_y_discrete(labels=(values=names.nit)) +
  labs(x= "Relative Abundance", y= "Nitrogen genes")+
  scale_x_continuous(labels = scientific)+ theme_bw() +
  geom_errorbar(aes(xmin=mean-sd, xmax=mean+sd), width=.2,position=position_dodge(.9)) +
  scale_fill_manual(values = (values=colour.nit),labels = labels.nit.cyc)+
#Significantly different methane groups plot
names.met = c("fwdA/fmdA","mtaC","mch","hdrA2","mtdB")
labels.nit.cyc = c("Exotic forest", "Grassland to exotic","Exotic to grassland","Grassland")
colour.nit = c("orangered1","gray60","gray46", "mediumseagreen")
```

```
methane.p = ggplot(meth.mean.sd, aes(x=mean, y=Gene, fill=LandUse)) +
  geom_bar(stat="identity", position=position_dodge()) +
  labs(x= "Relative Abundance", y= "Methane cycling genes")+
  scale_y_discrete(labels=(values=names.met)) +
  labs(x= "Relative Abundance", y= "Methane genes")+
  scale_x_continuous(labels = scientific)+ theme_bw() +
  geom_errorbar(aes(xmin=mean-sd, xmax=mean+sd), width=.2,position=position_dodge(.9)) +
  scale_fill_manual(values = (values=colour.nit),labels = labels.nit.cyc)
```
