# Supplementary material for: Land use modification causes slow, but predictable, change in soil microbial community composition and functional potential
Source: Environ Microbiome. 2023 Apr 6;18:30. doi: 10.1186/s40793-023-00485-x (PMC10080853; doi:10.1186/s40793-023-00485-x)
Supplement: Supplementary file 3 — Additional file 3. R code associated with taxonomic data processing. [file 40793_2023_485_MOESM3_ESM.pdf]

National_soils_code

2022-11-29

Packages

```
library(ggrepel)
library(vegan)
library(tidyverse)
library(phyloseq)
library(dada2)
library(ggplot2)
library(devtools)
library(knitr)
library(tidyverse)
library(propr)
library(vegan)
library(cowplot)
library(zoo)
library(tibble)
library(doParallel)
library(foreach)
library(phangorn)
library(reshape2)
library(reshape)
library(usedist)
library(FSA)
library(rcompanion)
```

Amplicon sequence pre-processing DADA2

```
##Set path from which to call raw fastq files
path2 = "./seperated_raw_samples"
list.files(path2)

##Extract sample names, assuming filenames have format: SAMPLENAME_XXX.fastq
f.names2 = as.vector(list.files(path2, pattern="_R1_001.fastq", full.names = F))
r.names2 = as.vector(list.files(path2, pattern="_R2_001.fastq", full.names = F))
fnFs2 = sort(list.files(path2, pattern="_R1_001.fastq", full.names = TRUE))
fnRs2 = sort(list.files(path2, pattern="_R2_001.fastq", full.names = TRUE))
list (fnFs2)
list (fnRs2)
##Plot quality scores
qpf = plotQualityProfile(fnFs2[1:6])
qpr = plotQualityProfile(fnRs2[1:6])
quartz(qpr)
##Filtering and trimming (quality control step)
filt_path2 = file.path(path2, "filtered") #place filtered files in "filtered" subdirectory
```

```r
filtFs2 = file.path(filt_path2, paste0(f.names2))
filtRs2 = file.path(filt_path2, paste0(r.names2))
##Remove primers
out2 = filterAndTrim(fnFs2, filtFs2, fnRs2, filtRs2, truncLen=c(270,240), trimLeft=c(17,21),
                     maxN=0, maxEE=c(4,4), truncQ=2, rm.phix=TRUE,
                     compress=TRUE, multithread=TRUE)
##Parsing
sample.names2 <- sapply(strsplit(basename(filtFs2), "_"), `[`, 1)
sample.namesR2 <- sapply(strsplit(basename(filtRs2), "_"), `[`, 1)
if(!identical(sample.names2, sample.namesR2)) stop("Forward and reverse files do not match.")
names(filtFs2) <- sample.names2
names(filtRs2) <- sample.names2
set.seed(100)
# Learn forward error rates
errF2 <- learnErrors(filtFs2, nbases=1e8, multithread=TRUE)
# Learn reverse error rates
errR2 <- learnErrors(filtRs2, nbases=1e8, multithread=TRUE)
# Sample inference and merger of paired-end reads
mergers2 <- vector("list", length(sample.names2))
names(mergers2) <- sample.names2
for(sam in sample.names2) {
  cat("Processing:", sam, "\n")
  derepF2 <- derepFastq(filtFs2[[sam]])
  ddF2 <- dada(derepF2, err=errF2, multithread=TRUE)
  derepR2 <- derepFastq(filtRs2[[sam]])
  ddR2 <- dada(derepR2, err=errR2, multithread=TRUE)
  merger2 <- mergePairs(ddF2, derepF2, ddR2, derepR2)
  mergers2[[sam]] <- merger2
}

# Construct sequence table and remove chimeras
seqtab2 <- makeSequenceTable(mergers2)
dim(seqtab2)
table(nchar(getSequences(seqtab2)))
#Remove chimeras
seqtab.ex.chi2 = removeBimeraDenovo(seqtab2, method = 'consensus',
                                    multithread = T, verbose= T)
dim(seqtab.ex.chi2)
row.names(seqtab.ex.chi2)
###Calculation proportion of variants identified as chimeras
sum(seqtab.ex.chi2)/sum(seqtab2)
# Track
getN <- function(x) sum(getUniques(x))
track2 <- cbind(out2,sapply(mergers2, getN), rowSums(seqtab.ex.chi2),
          round(rowSums(seqtab.ex.chi2)/out2[,2]*100, 1),
          round(rowSums(seqtab.ex.chi2)/out2[,1]*100, 1))
colnames(track2) <- c("input", "filtered", "mergers", "nonchim",
                      "perc of filt", "final perc")
rownames(track2) <- sample.names2
head(track2)

###Assign taxonomy####
dna <- DNAStringSet(getSequences(seqtab.ex.chi2)) # Create a DNAStringSet from the ASVs
```

```
load("~/Desktop/National_soils/Land_use/SILVA.RData")
ids <- IdTaxa(dna, trainingSet, strand="top", processors=NULL, verbose=FALSE)
ranks <- c("domain", "phylum", "class", "order", "family", "genus", "species")
taxid <- t(sapply(ids, function(x) {
  m <- match(ranks, x$rank)
  taxa <- x$taxon[m]
  taxa[startsWith(taxa, "unclassified_")] <- NA
  taxa
}))
colnames(taxid) <- ranks; rownames(taxid) <- getSequences(seqtab)
taxa <- taxid
taxa[taxa == " "] <- ""

##Format metadata so all sorted alphabetically in same order
met <- read.table("./met_16S_redo.txt", sep="\t", header=T, row.names = 1)
met_ordered = met[order(row.names(met)),]##Sort by site alphabeticaly
seq_ordered = seqtab[order(row.names(seqtab)),]
##Make names same for seqtable as mapping file
sample.rows <- rownames(met_ordered)
rownames(seq_ordered) <- sample.rows

##Construct phyloseq object
ps <- phyloseq(otu_table(seq_ordered, taxa_are_rows=FALSE), sample_data(met_ordered),
               tax_table(taxa))
#Reformat
dna2 <- Biostrings::DNAStringSet(taxa_names(ps))
names(dna2) <- taxa_names(ps)
ps <- merge_phyloseq(ps, dna2)
taxa_names(ps) <- paste0("ASV", seq(ntaxa(ps)))
```

Merge replicates function

```
##Merge replicates####

# Function to merge samples by averaging OTU counts instead of summing
merge_samples_mean <- function(physeq, group, round = "floor"){
  # Calculate the number of samples in each group
  group_sums <- as.matrix(table(sample_data(physeq)[ ,group]))[,1]

  # Merge samples by summing
  merged <- merge_samples(physeq, group)

  # Divide summed OTU counts by number of samples in each group
  x <- as.matrix(otu_table(merged))
  if(taxa_are_rows(merged)){ x<-t(x) }

  # Pick the rounding functions
  if (round == "floor"){
    out <- floor(x/group_sums)
  } else if (round == "round"){
    out <- myround(x/group_sums) ##(t(x/group_sums))
  }
```

```
  # Return new phyloseq object with taxa as rows
  out <- otu_table(out, taxa_are_rows = FALSE)##TRUE if taxa as rows
  otu_table(merged) <- out
  return(merged)
}
```

Merging samples

```
#Merge samples by replicates
ps.merged = merge_samples_mean(ps, "Site")
#View how many reads per sample
sums.2 = sample_sums(otu_table(ps.merged))
##Check that merging gives mean
otu_table(ps)[1:10,1:5]
otu_table(ps.merged)[1:20,1:5]
##Merge with mapping file
sample_data(ps.merged) <- meta  ##add new mapping file to phyloseq object
#Remove singletons
ps.merged.filt <- filter_taxa(ps.merged, function (x) {sum(x > 0) > 1}, prune=TRUE)
##Filter taxa####
merged.bac = subset_taxa(ps.merged.filt, domain=="Bacteria")
merged.bac = subset_taxa(merged.bac, order!="Chloroplast")
merged.bac = subset_taxa(merged.bac, family!="Mitochondria")
###Rarefaction Curve
gt.curve = ggrare(ps.merged.filt, step = 350, color = "LandUse", se = FALSE)
colours.rare = c("orangered1","mediumseagreen","mediumpurple3","cornflowerblue")
gt.curve  + theme_bw() + facet_wrap(~LandUse) +
  theme(axis.title.y = element_text(size = 11),
        legend.text = element_text(size = 8), axis.text.x = element_text(size = 8),
        axis.text.y = element_text(size = 8), strip.text = element_text(size = 10),
        strip.background = element_blank()) +
  scale_color_manual(values = (values=colours.rare))
#Rarefaciton curve cut off at 4500
rare.labels = c(Exotic = "Exotic forest",Grassland = "Grassland",
                Horticulture = "Horticulture",Indigenous ="Indigenous")
gt.curve  + theme_bw() + facet_wrap(~LandUse, labeller = as_labeller(rare.labels)) +
  theme(axis.title.y = element_text(size = 11), axis.text.x = element_text(size = 8),
        axis.text.y = element_text(size = 8), strip.text = element_text(size = 10),
        strip.background = element_blank(), panel.grid.major = element_blank(),
        panel.grid.minor = element_blank(), legend.position = "none",
panel.background = element_blank(), axis.line = element_line(colour = "black")) +
  scale_color_manual(values = (values=colours.rare)) + xlim(0,5000) + ylim(0,1500)
##Rarefy data to minumum sample depth
set.seed(500)
sums = sample_sums(otu_table(merged.bac))
min_sum = min(sums)
ps.rare = rarefy_even_depth(merged.bac, sample.size = min_sum)
```

CSS normalisation

```
ps.filt = subset_taxa(ps.merged, domain=="Bacteria")
ps.filt = subset_taxa(ps.filt, order!="Chloroplast")
merged.bac = subset_taxa(merged.bac, family!="Mitochondria")
```

```
pruned.all<-filter_taxa(ps.filt, function(x) sum(x) > 2, TRUE) #remove singletons

MGS <- phyloseq_to_metagenomeSeq(pruned.all)
p <- cumNormStatFast(MGS)
p #0.5
MGS.norm.all <- cumNorm(MGS, p =p)
norm.all <- MRcounts(MGS.norm.all, norm = TRUE, log = FALSE)
data_norm.all <- pruned.all
otu_table(data_norm.all) <- t(otu_table(norm.all, taxa_are_rows = T))

# CSS will sometimes decrease the fold-difference in sampling depth but not always.
max(sample_sums(ps.filt))/min(sample_sums(ps.filt))
max(sample_sums(pruned.all))/min(sample_sums(pruned.all))
max(sample_sums(data_norm.all))/min(sample_sums(data_norm.all))
data_norm_RA.all<-transform_sample_counts(data_norm.all, function(x) x / sum(x) )
```

Subsetting for each transition and their long-term counterparts

```
ps.16S = data_norm_RA.all
ps.GE.longterm = subset_samples(data_norm_RA.all, Transition_spec == "G->E" |
                                 Transition_spec == "Grassland" |
                                 Transition_spec == "Exotic")
ps.GH.longterm = subset_samples(data_norm_RA.all, Transition_spec == "G->H" |
                                 Transition_spec == "Grassland" |
                                 Transition_spec == "Horticulture")
ps.EG.longterm = subset_samples(data_norm_RA.all, Transition_spec == "E->G" |
                                 Transition_spec == "Exotic" |
                                 Transition_spec == "Grassland")
bac.RA.phyla <- tax_glom(ps.16S, taxrank = "phylum")
bac.RA.phyla = transpose_otu_table(bac.RA.phyla)
```

#Alpha diversity

```
richness = estimate_richness(ps.rare, measures = c( "InvSimpson", "Chao1"))
met.2 = as.data.frame(sample_data(ps.rare))
met.richness <- as.data.frame(cbind(richness,met.2))

#Dunn test Chao1
alpha.dunn = dunnTest(Chao1 ~ Clean_transition,
                      data=met.richness,method="holm")
alpha.dunn2= alpha.dunn$res
alpha.letters = cldList(P.adj ~ Comparison, data=alpha.dunn2,threshold  = 0.05)
alpha.letters$Clean_names = c("Exotic","Exotic to Grassland",
                              "Grassland", "Grassland to Exotic",
                              "Grassland to Horticulture", "Horticulture",
                              "Indigenous")
#Significance letters - no significant difference for Chao1
Clean_names = c("Exotic","Exotic to Grassland", "Grassland",
                "Grassland to Exotic","Grassland to Horticulture",
                "Horticulture","Indigenous")
Letter <- c("a", "a","a","a", "a","a","a")
Chao1.letter = data.frame(Clean_names, Letter)
Chao1.letter$Site_type = c("Representative","Transitional",
```

```
                           "Representative","Transitional",
                           "Transitional","Representative",
                           "Representative")
ggplot(met.richness, aes(x = Clean_names, y = Chao1,  fill= Clean_names)) +
  geom_boxplot(aes(fill=Clean_names)) + theme_bw()+
  theme(panel.grid.major = element_blank(), panel.grid.minor = element_blank(),
       legend.position = "none", axis.text.x = element_text(angle = 55, hjust=1, size = 11),
       axis.title=element_text(size=12))+   geom_jitter(width = 0.2,  alpha =0.8)+
  theme(strip.background = element_blank(),strip.text.x = element_text(size=12))+
  labs(y = "Chao1 Index", fill = "Land use", x= " ") +
  geom_text(data=Chao1.letter, aes(label = Letter, x = Clean_names, y = 4100), size = 5)+
  scale_fill_manual(values = colours.alpha)


#Dunn test Inverse Simpson
alpha.dunn = dunnTest(InvSimpson ~ Clean_transition,
                      data=met.richness,method="holm")
alpha.dunn2= alpha.dunn$res
alpha.letters = cldList(P.adj ~ Comparison, data=alpha.dunn2,threshold  = 0.05)
alpha.letters$Clean_names = c("Exotic","Exotic to Grassland",
                              "Grassland", "Grassland to Exotic",
                              "Grassland to Horticulture",
                              "Horticulture","Indigenous")
#Add landuse for x axis placement
alpha.letters$Site_type = c("Representative","Transitional",
                            "Representative","Transitional","Transitional",
                            "Representative","Representative")
#Add colours in order of levels
levels(met.richness$Clean_names)
colours.alpha = c("orangered1","grey","mediumseagreen","grey",
                  "mediumpurple3","grey","cornflowerblue")

ggplot(met.richness, aes(x = Clean_names, y = InvSimpson,  fill= Clean_names)) +
  geom_boxplot(aes(fill=Clean_names)) + theme_bw()+
  theme(panel.grid.major = element_blank(), panel.grid.minor = element_blank(),
       legend.position = "none", axis.text.x = element_text(angle = 55, hjust=1, size = 11),
       axis.title=element_text(size=12))+geom_jitter(width = 0.2,  alpha =0.8)+
  theme(strip.background = element_blank(),strip.text.x = element_text(size=12))+
  labs(y = "Inverse Simpson Index", fill = "Land use", x= " ") +
  geom_text(data=alpha.letters, aes(label = Letter, x = Clean_names, y = 700), size = 5) +
  scale_fill_manual(values = colours.alpha)
```

NMDS with environmental vectors

```
otu = as(otu_table(data_norm_RA.all),"matrix")
# transpose if necessary
if(taxa_are_rows(data_norm_RA.all)){otu <- t(otu)}
# Coerce to data.frame
otu.df = as.data.frame(otu)
otu.df = otu.df[order(row.names(otu.df)),]##Sort by site alphabeticaly
meta.67 = meta.67[order(row.names(meta.67)),]##Sort by site alphabeticaly
MDS<-metaMDS(otu, distance="bray", k=2, trymax=999, autotransform = FALSE)
MDS_points<-data.frame(MDS$points)
```

```
MDS_map<-cbind(MDS_points,meta.67)
identical(rownames(otu.df), rownames(meta.67))

chem.stand<-decostand(meta.67[,15:26], margin=2, method="standardize", na.rm=T)
names(chem.stand)<-c("pH", "TN", "OlsenP*", "CtoN", "NO3-N", "NH4-N*",
                     "MP", "Arsenic*","Cadmium*", "Chromium*", "Copper*", "Zinc")
fit <- envfit(MDS, chem.stand, perm = 999, na.rm=T)
scores(fit, "vectors")
env.scrs <- as.data.frame(scores(fit, display = "vectors"))
env.scrs <- cbind(env.scrs, Variable = rownames(env.scrs))

##PERMANOVA
data_norm_RA.all.sd = as(sample_data(data_norm_RA.all), "data.frame")
data_norm_RA.all.dist = phyloseq::distance(data_norm_RA.all, "bray")
data_norm_RA.all.sd = as(sample_data(data_norm_RA.all), "data.frame")
adonis2(data_norm_RA.all.dist ~ Clean_transition, data = data_norm_RA.all.sd)

levels(MDS_map$Clean_names)
MDS_map$Clean_names <- factor(MDS_map$Clean_names,
                              levels = c("Exotic","Grassland to Exotic",
                                         "Grassland","Exotic to Grassland",
                                         "Horticulture",
                                         "Grassland to Horticulture","Indigenous"))
ggplot(MDS_map, aes(MDS1, MDS2)) +
  geom_point(aes(shape = Clean_names, color = Clean_names), size=2) +
  scale_color_manual(values=(values=colours.merged)) + labs(fill="Land use") +
  scale_shape_manual(values = c(16,24,16,24,16,24,16,24))+
  geom_segment(data = env.scrs,
               aes(x = 0, xend = NMDS1/2, y = 0, yend = NMDS2/2),
               arrow = arrow(length = unit(0.25, "cm")), colour = "gray", size=0.5) +
  annotate("text", x=0.86, y=0.75, label= "P = 0.001") +
  annotate("text", x=0.8, y=0.85, label= "stress = 0.14") +
  geom_text_repel(data = env.scrs, aes(x = NMDS1/2, y = NMDS2/2, label = Variable),
                  alpha = 0.8, size = 4) +
  theme_bw() +  labs(y = "NMDS2", x= "NMDS1", colour = "Land Use", shape = "Land Use")+
  theme(panel.grid.major = element_blank(), panel.grid.minor = element_blank(),
        legend.text=element_text(size=10), legend.title= element_text(),
        legend.background = element_rect(color="grey50", size=.3, linetype=1))
```

Single axis plots

```
#Single axis NMDS points Grassland to Exotic
MDS.GE = ordinate(ps.GE.longterm, method = "NMDS", distance = "bray",
                  trymax = 999, autotransform = F)
MDS.points.GE=data.frame(MDS.GE$points)
met.GE = as.data.frame(sample_data(ps.GE.longterm))
MDS.GE.df<-cbind(MDS.points.GE,met.GE)
MDS.GE.df[,'Transition.age']<-factor(MDS.GE.df[,'Transition.age'])
MDS.GE.df =MDS.GE.df %>%
  mutate(Transition.age = coalesce(Transition.age, Transition_spec))

#Dunn's test EG
pt = dunnTest(MDS1 ~ Transition.age, data=MDS.GE.df, method = "holm")
```

```
PT3= pt$res
GE.mds.letters = cldList(P.adj ~ Comparison, data=PT3,threshold  = 0.05)

levels(MDS.GE.df$Transition.age)
MDS.GE.df$Transition.age <- factor(MDS.GE.df$Transition.age,
                                   levels = c("Grassland","Recent",
                                              "Historic","Exotic"))
ggplot(data = MDS.GE.df, aes(Transition.age,MDS1)) +
  geom_point(aes(colour = Transition.age ), size=2) +
  scale_color_manual(values=c("mediumseagreen","dimgrey","darkgrey","orangered1"))+
  theme_bw() + labs(colour = "Land Use")+ theme(axis.title.x=element_blank()) +
  geom_text(data=GE.mds.letters, aes(label = Letter, x = Group, y = 1), size = 5) +
  labs(y = "NMDS1") +
  theme(panel.grid.major = element_blank(), panel.grid.minor = element_blank(),
       legend.position = "none", axis.text.x = element_text(size = 11),
       axis.title=element_text(size=12))

#Single axis NMDS points Grassland to Horticulture
MDS.GH = ordinate(ps.GH.longterm, method = "NMDS", distance = "bray",
                  trymax = 999, autotransform = F)
MDS.points.GH=data.frame(MDS.GH$points)
met.GH = as.data.frame(sample_data(ps.GH.longterm))
MDS.GH.df<-cbind(MDS.points.GH,met.GH)
MDS.GH.df[,'Transition.age']<-factor(MDS.GH.df[,'Transition.age'])
MDS.GH.df =MDS.GH.df %>%
  mutate(Transition.age = coalesce(Transition.age, Transition_spec))

#Dunn's test GH
pt = dunnTest(MDS1 ~ Transition.age, data=MDS.GH.df, method = "holm")
PT3= pt$res
GH.mds.letters = cldList(P.adj ~ Comparison, data=PT3,threshold  = 0.05)

levels(MDS.GH.df$Transition.age)
MDS.GH.df$Transition.age <- factor(MDS.GH.df$Transition.age,
                                   levels = c("Grassland","Recent",
                                              "Historic","Horticulture"))

ggplot(data = MDS.GH.df, aes(Transition.age,MDS1)) +
  geom_point(aes(colour = Transition.age ), size=2) +
  scale_color_manual(values=c("mediumseagreen","dimgrey","darkgrey","mediumpurple3"))+
  theme_bw() + labs(colour = "Land Use")+ theme(axis.title.x=element_blank()) +
  geom_text(data=GH.mds.letters, aes(label = Letter, x = Group, y = 1), size = 5) +
  labs(y = "NMDS1") +
  theme(panel.grid.major = element_blank(), panel.grid.minor = element_blank(),
       legend.position = "none", axis.text.x = element_text(size = 11),
       axis.title=element_text(size=12))

#Single axis NMDS points Exotic to Grassland
MDS.EG = ordinate(ps.EG.longterm, method = "NMDS", distance = "bray",
                  trymax = 999, autotransform = F)
MDS.points.EG=data.frame(MDS.EG$points)
met.EG = as.data.frame(sample_data(ps.EG.longterm))
MDS.EG.df<-cbind(MDS.points.EG,met.EG)
```

```r
MDS.EG.df[,'Transition.age']<-factor(MDS.EG.df[,'Transition.age'])
MDS.EG.df =MDS.EG.df %>%
  mutate(Transition.age = coalesce(Transition.age, Transition_spec))

#Dunn's test EG
pt = dunnTest(MDS1 ~ Transition.age, data=MDS.EG.df, method = "holm")
PT3= pt$res
EG.mds.letters = cldList(P.adj ~ Comparison, data=PT3,threshold  = 0.05)

levels(MDS.EG.df$Transition.age)
MDS.EG.df$Transition.age <- factor(MDS.EG.df$Transition.age,
                                   levels = c("Exotic","Recent",
                                              "Historic","Grassland"))
ggplot(data = MDS.EG.df, aes(Transition.age,MDS1)) +
  geom_point(aes(colour = Transition.age ), size=2) +
  scale_color_manual(values=c("orangered1","dimgrey","darkgrey","mediumseagreen"))+
  theme_bw() + labs(colour = "Land Use")+ theme(axis.title.x=element_blank()) +
  geom_text(data=EG.mds.letters, aes(label = Letter, x = Group, y = 1), size = 5) +
  labs(y = "NMDS1") +
  theme(panel.grid.major = element_blank(), panel.grid.minor = element_blank(),
       legend.position = "none", axis.text.x = element_text(size = 11),
       axis.title=element_text(size=12))
```

MW test for differential phyla

```r
#Subset samples by land use
ps.exotic.grassland = subset_samples(bac.RA.phyla, Transition_spec == "Exotic" |
                                       Transition_spec == "Grassland")
ps.grassland.horticulture = subset_samples(bac.RA.phyla, Transition_spec == "Grassland" |
                                             Transition_spec == "Horticulture")

library(broom)
RANK = 'phylum'
dat <- ps.exotic.grassland %>%
  tax_glom(taxrank = RANK) %>%
  psmelt()

#Test whether relative abundance differs between long-term sites
dat %>%
  group_by_(RANK) %>%
  do(tidy(kruskal.test(Abundance ~ Transition_spec, data=., exact=F))) %>%
  ungroup() %>%
  mutate(p.adjust=p.adjust(p.value)) -> mw.results
#Summarize results
mw.results %>%
  subset(p.value < 0.05) %>%
  knitr::kable()
#Extract significant values
mw.grassland.exotic = mw.results %>%
  subset(p.value < 0.05)
mw.nitrogen
mw.sulfur
```

```r
mean_PGroup = sapply(levels(SampleType),function(i){
  rowMeans(otu_table(PGroup)[,SampleType==i])
})
```

Dominant phyla bar plots

```r
#Subset by taxa that significantly differed between long-term land uses
ps.GE.lt.phyla= subset_samples(bac.RA.phyla, Transition_spec == "G->E" |
                                Transition_spec == "Grassland" |
                                Transition_spec == "Exotic")
ps.GE.lt.phyla.sig = subset_taxa(ps.GE.lt.phyla, phylum=="Acidobacteriota"|
                                  phylum=="Actinobacteriota"| phylum=="Firmicutes"|
                                  phylum=="Myxococcota"| phylum== "Proteobacteria"|
                                  phylum=="Verrucomicrobiota")

ge.df = df[,c("Abundance","Transition_spec","phylum")]  # returns a data.frame
library(dplyr)
grouped <- group_by(ge.df, Transition_spec, phylum)
ge.sum = summarise(grouped, mean=mean(Abundance), se=se(Abundance))
labels.met.GE = c("Exotic forest","Grassland to exotic forest","Grassland")
colours.met = c("orangered1", "gray27", "mediumseagreen")

phy.bar.ge = ggplot(data = ge.sum, aes(x=mean, y=phylum, fill=Transition_spec)) +
  geom_bar(stat="identity", position=position_dodge()) +
  scale_y_discrete(labels=(values=ge.sum)) +
  labs(x= "Relative Abundance", y= "Abundant phyla")+
    scale_x_continuous(labels = scales::percent_format(accuracy = 5L))+
  geom_errorbar(aes(xmin=mean-se, xmax=mean+se), width=.2,
                position=position_dodge(.9)) + theme_bw() +
  scale_fill_manual(values = (values=colours.met),labels = labels.met.GE)+
  theme(panel.grid.major = element_blank(), panel.grid.minor = element_blank(),
        panel.border = element_rect(linetype = "solid", colour = "black", size=0.9),
        legend.title = element_blank(),
        axis.text= element_text(colour="black",size=20),
        legend.position = c(0.745, 0.9),axis.title=element_text(size=22),
        legend.text=element_text(size=20),
        axis.ticks.y=element_blank(),plot.title = element_text(hjust = 0.5, size = 25),
        axis.title.x = element_blank()) +
  ggtitle(expression(paste( "Grassland" %->% " exotic forest")))

ps.GH.lt.phyla = subset_samples(bac.RA.phyla, Transition_spec == "G->H" |
                                 Transition_spec == "Grassland" |
                                 Transition_spec == "Horticulture")
ps.GH.lt.phyla.sig = subset_taxa(ps.GH.lt.phyla, phylum=="Firmicutes"|
                                  phylum=="Myxococcota"| phylum== "Proteobacteria"|
                                  phylum=="Verrucomicrobiota"| phylum=="Nitrospirota")
ps.GH.melt = psmelt(ps.GH.lt.phyla.sig)

gh.df = ps.GH.melt[,c("Abundance","Transition_spec","phylum")]
grouped.gh <- group_by(gh.df, Transition_spec, phylum)
gh.sum = summarise(grouped.gh, mean=mean(Abundance), se=se(Abundance))
levels(gh.sum$Transition_spec)
gh.sum$Transition_spec <- factor(gh.sum$Transition_spec,
```

```
                                 levels = c( "Horticulture","G->H","Grassland"))
labels.GH = c("Horticulture","Grassland to horticulture","Grassland")
colours.GH = c("mediumpurple3", "gray27", "mediumseagreen")

phy.bar.gh = ggplot(data = gh.sum, aes(x=mean, y=phylum, fill=Transition_spec)) +
  geom_bar(stat="identity", position=position_dodge()) +
  scale_y_discrete(labels=(values=ge.sum)) +labs(x= "Relative Abundance",
                                                 y= "Abundant phyla")+
    scale_x_continuous(labels = scales::percent_format(accuracy = 5L))+
  geom_errorbar(aes(xmin=mean-se, xmax=mean+se), width=.2,
                position=position_dodge(.9)) + theme_bw() +
  scale_fill_manual(values = (values=colours.GH),labels = labels.GH)+
  theme(panel.grid.major = element_blank(), panel.grid.minor = element_blank(),
        panel.border = element_rect(linetype = "solid",
                                    colour = "black", size=0.9),
        legend.title = element_blank(),
        axis.text= element_text(colour="black",size=20),
        legend.position = c(0.75, 0.9),
        axis.title=element_text(size=22),legend.text=element_text(size=20),
        axis.ticks.y=element_blank(),plot.title = element_text(hjust = 0.5, size = 25),
        axis.title.x = element_blank()) +
  ggtitle(expression(paste( "Grassland" %->% " horticulture")))


ps.EG.lt.phyla = subset_samples(bac.RA.phyla, Transition_spec == "E->G" |
                                  Transition_spec == "Exotic" |
                                  Transition_spec == "Grassland")
ps.EG.lt.phyla.sig = subset_taxa(ps.EG.lt.phyla, phylum=="Acidobacteriota"|
                                   phylum=="Actinobacteriota"| phylum=="Firmicutes"|
                                   phylum=="Myxococcota"| phylum== "Proteobacteria"|
                                   phylum=="Verrucomicrobiota")
ps.EG.melt = psmelt(ps.EG.lt.phyla.sig)
eg.df = ps.EG.melt[,c("Abundance","Transition_spec","phylum")]  # returns a data.frame
grouped.eg <- group_by(eg.df, Transition_spec, phylum)
eg.sum = summarise(grouped.eg, mean=mean(Abundance), se=se(Abundance))

levels(eg.sum$Transition_spec)
eg.sum$Transition_spec <- factor(eg.sum$Transition_spec,
                                 levels = c( "Grassland","E->G","Exotic"))
labels.EG = c("Grassland","Exotic forest to grassland","Exotic forest")
colours.EG = c("mediumseagreen","gray27","orangered1")
phy.bar.eg = ggplot(data = eg.sum, aes(x=mean, y=phylum, fill=Transition_spec)) +
  geom_bar(stat="identity", position=position_dodge()) +
  scale_y_discrete(labels=(values=ge.sum)) +
  labs(x= "Relative Abundance",y= "Abundant phyla")+
    scale_x_continuous(labels = scales::percent_format(accuracy = 5L))+
  geom_errorbar(aes(xmin=mean-se, xmax=mean+se), width=.2,
                position=position_dodge(.9)) + theme_bw() +
  scale_fill_manual(values = (values=colours.EG),labels = labels.EG)+
  theme(panel.grid.major = element_blank(), panel.grid.minor = element_blank(),
        panel.border = element_rect(linetype = "solid", colour = "black", size=0.9),
        legend.title = element_blank(), axis.text= element_text(colour="black",size=20),
        legend.position = c(0.75, 0.9),axis.title=element_text(size=22),
```

```
        legend.text=element_text(size=20), axis.ticks.y=element_blank(),
        plot.title = element_text(hjust = 0.5, size = 25)) +
  ggtitle(expression(paste( "Exotic forest" %->% " grassland")))
```

Indicator species

```
library(indicspecies)
library(plyr)
library(labdsv)
ps.16S.tran = subset_samples(ps.16S, Site_type == "Transitional")
ps.16S.rep = subset_samples(ps.16S, Site_type == "Representative")
#Extract OTU tables from each ps object
OTU1 = as(otu_table(ps.16S.tran), "matrix")
# transpose if necessary
if(taxa_are_rows(ps.16S.tran)){OTU1 <- t(OTU1)}
# Coerce to data.frame
rep.otu.df = as.data.frame(OTU1)
tran.otu.df = as.data.frame(OTU1)
#Remove zeros
rep.otu.df.zero = rep.otu.df[, !apply(rep.otu.df==0,2,all)]
tran.otu.df.zero = tran.otu.df[, !apply(tran.otu.df==0,2,all)]
#Get mapping files
rep.met = as(sample_data(ps.16S.rep), "data.frame")
tran.met = as(sample_data(ps.16S.tran), "data.frame")
identical(row.names(tran.met), rownames(tran.otu.df))
#Extract land uses for each and assign a numerical value
landuse <- rep.met[,3, drop=FALSE]
landuse$value <- revalue(landuse$Transition,
               c("Exotic"="1", "Grassland"="2", "Horticulture"="3", "Indigenous"="4"))
landuse.tran = tran.met$Transition
#Run indval analysis for multiple groups
(iva2 <- multipatt(rep.otu.df.zero,grps,max.order = 2,control = how(nperm = 999)))
(iva.tran <- multipatt(tran.otu.df.zero,landuse.tran,max.order = 2,
                       control = how(nperm = 999)))
# Export the result to file
options(max.print = 200)
capture.output(summary(iva2, indvalcomp = TRUE), file="indicsummary_representative")
capture.output(summary(iva.tran, indvalcomp = TRUE), file="indicsummary_transitional")
#Repeat for sites split by transition history
history.tran = tran.met$Transition_history
(iva.hist <- multipatt(tran.otu.df.zero,history.tran,max.order = 2,
                       control = how(nperm = 999)))
capture.output(summary(iva.hist, indvalcomp = TRUE), file="indicsummary_history")

#Subsetting indicator data
ind.combined <- read.table("indicator.combined.txt", sep='\t',
                           header=TRUE,  stringsAsFactors = TRUE)
ind.history <- read.table("indicator.history.txt", sep='\t',
                          header=TRUE,  stringsAsFactors = TRUE)
library(eulerr)
set.seed(1)
citation("eulerr")
exotic.venn = subset(ind.combined, Landuse == "Exotic")
```

```r
grass.venn = subset(ind.combined, Landuse == "Grassland")
hort.venn = subset(ind.combined, Landuse == "Horticulture")
EG.venn = subset(ind.combined, Landuse == "E->G")
GE.venn = subset(ind.combined, Landuse == "G->E")
ExoticGrass.venn = subset(ind.combined, Landuse == "ExoticGrass")
#Subset by historic split
EG.h= subset(ind.history,Landuse == "Historic_E->G")
EG.r=subset(ind.history,Landuse == "Recent_E->G")
GE.h= subset(ind.history,Landuse == "Historic_G->E")
GE.r=subset(ind.history,Landuse == "Recent_G->E")
GH.h= subset(ind.history,Landuse == "Historic_G->H")
GH.r=subset(ind.history,Landuse == "Recent_G->H")
```

Venn diagrams of indicator taxa

```r
#Exotic to grassland
#Colours + labels
cols.2 <- c(Exotic = "orangered1", Grassland = "mediumseagreen",
            EG.h.v = "gray54",EG.r.v = "gray72")
labs.eg.2 <- c(Exotic = "Exotic", Grassland = "Grassland", EG.h.v = "Historic")
labs.eg.3 <- c(Exotic = "Exotic", Grassland = "Grassland", EG.r.v = "Recent")

#Historic
eg.2<- list(Exotic=exotic.venn$ASV,
           Grassland=grass.venn$ASV,
           EG.h.v=EG.h$ASV)
labs.2 <- c(Exotic = "Exotic forest", Grassland = "Grassland",
            EG.h.v = "Historic",EG.r.v = "Recent")
venn.eg.historic = plot(euler(s2), quantities = list(cex = 1.2),
    shape = "ellipse",
     alpha = 0.6, fills = cols.2,labels = list(labels = labs.2,fontsize =18),
     edges = cols.2)
venn.eg.historic
#Recent
cols.3 <- c(Exotic = "orangered1", Grassland = "mediumseagreen", EG.r.v = "gray72")
labs.3 <- c(Exotic = "Exotic forest", Grassland = "Grassland",EG.r.v = "Recent")
eg.3<- list(Exotic=exotic.venn$ASV,
           Grassland=grass.venn$ASV,
           EG.r.v=EG.r$ASV)
venn.eg.recent = plot(euler(eg.3), quantities = list(cex = 1.2),
      shape = "ellipse",
     alpha = 0.6, fills = cols.3,labels = list(labels = labs.3,fontsize =18 ),
     edges = cols.3)
venn.eg.recent

#Grassland to exotic
#Colours + labels
cols.4 <- c(Grassland = "mediumseagreen", Exotic = "orangered1",
            ge.h.v = "gray54",ge.r.v = "gray72")
labs.4 <- c(Grassland = "Grassland",Exotic = "Exotic",
            ge.h.v = "Historic",ge.r.v = "Recent")
#Historic
ge.2<- list(Grassland=grass.venn$ASV,
```

```
            ge.h.v=GE.h$ASV,
           Exotic=exotic.venn$ASV )
cols.ge.2 <- c("mediumseagreen","gray54", "orangered1" )
labs.ge.2 <- c( Grassland = "Grassland",ge.r.v = "Historic",
                Exotic = "Exotic forest")
venn.ge.historic = plot(euler(ge.2), quantities = list(cex = 1.5),
      shape = "ellipse",
     alpha = 0.6, fills = cols.ge.2,labels = list(labels = labs.ge.2,
                                                  fontsize =18 ),
     edges = cols.ge.2)
venn.ge.historic
#Recent
ge.3<- list(Grassland=grass.venn$ASV,
            ge.r.v=GE.r$ASV,
            Exotic=exotic.venn$ASV )
cols.ge.3 <- c("mediumseagreen","gray72", "orangered1")
labs.ge.3 <- c("Grassland","Recent","Exotic forest")
venn.ge.recent = plot(euler(ge.3), quantities = list(cex = 1.5),
     shape = "ellipse",
     alpha = 0.6, fills = cols.ge.3,labels = list(labels = labs.ge.3,
                                                  fontsize =18 ),
     edges = cols.ge.3,  main = list(label = "Taxonomic", cex = 2))
venn.ge.recent

#Grassland to horticulture
#Historic
gh.2<- list(Grassland=grass.venn$ASV,
           gh.h.v=GH.h$ASV,
          Horticulture=hort.venn$ASV)
cols.gh.2 <- c("mediumseagreen","gray54","mediumpurple3" )
labs.gh.2 <- c("Grassland","Historic","Horticulture")
venn.gh.historic = plot(euler(gh.2), quantities = list(cex = 1.2),
     shape = "ellipse",
     alpha = 0.6, fills = cols.gh.2,labels = list(labels = labs.gh.2,
                                                  fontsize =18),
     edges = cols.gh.2)
venn.gh.historic
ggsave("venn.gh.historic.16S.png", venn.gh.historic)
#Recent
gh.3<- list(Grassland=grass.venn$ASV,
          gh.r.V=GH.r$ASV,
          Horticulture=hort.venn$ASV)
cols.gh.3 <- c("mediumseagreen", "gray72","mediumpurple3" )
labs.gh.3 <- c("Grassland", "Recent", "Horticulture")
venn.gh.recent = plot(euler(gh.3), quantities = list(cex = 1.2),
     shape = "ellipse",
     alpha = 0.6, fills = cols.gh.3,labels = list(labels = labs.gh.3,
                                                  fontsize =18),
     main = list(label = "Taxonomic", cex = 2), edges = cols.gh.3)
venn.gh.recent

venn.gh.recent.kegg = plot(euler(gh.3.kegg), quantities = list(cex = 1.2),
     shape = "ellipse",
```

```
     alpha = 0.6, fills = cols.gh.3,labels = list(labels = labs.gh.3,
                                                  fontsize =18),
     main = list(label = "Functional", cex = 2),edges = cols.gh.3)
venn.gh.recent.kegg
ggsave("venn.gh.recent.16S.png", venn.gh.recent)
```

Indicator bar plots

```
library(scales)
library("RColorBrewer")
#Subset by shared indicator functions
shared.taxa.names <- read.table("shared_taxa_names.txt", sep='\t', header=TRUE)
as(shared.taxa.names$ASV, 'character')
#Subset file by transition
ind.GE = subset(shared.taxa.names, shared.taxa.names$Transition == "GE")
ind.GH = subset(shared.taxa.names, shared.taxa.names$Transition == "GH")
ind.EG = subset(shared.taxa.names, shared.taxa.names$Transition == "EG")
#Subset ps object by transition type
GE.merged = subset_samples(merged.bac, Transition_spec == "G->E" |
                             Transition_spec == "Exotic" |
                             Transition_spec == "Grassland")
GH.merged = subset_samples(merged.bac, Transition_spec == "G->H" |
                             Transition_spec == "Horticulture" |
                             Transition_spec == "Grassland")
EG.merged = subset_samples(merged.bac, Transition_spec == "E->G" |
                             Transition_spec == "Exotic" |
                             Transition_spec == "Grassland")
#Prune ps object by indicators
asv.GE = ind.GE$ASV
shared.GE <- prune_taxa(asv.GE, GE.merged)
asv.GH = ind.GH$ASV
shared.GH <- prune_taxa(asv.GH, GH.merged)
asv.EG = ind.EG$ASV
shared.EG <- prune_taxa(asv.EG, EG.merged)


ge.lables.bar = c("Longterm_Grassland"= "Grassland","Recent_G->E"= "Recent",
                  "Historic_G->E"="Historic",
                  "Longterm_Exotic"="Exotic")
levels(sample_data(shared.GE)$Transition_history)
sample_data(shared.GE)$Transition_history <-
  factor(sample_data(shared.GE)$Transition_history,
         levels = c("Longterm_Grassland","Recent_G->E","Historic_G->E",
                    "Longterm_Exotic"))

plot_bar(shared.GE, fill="taxa_ASV") +
  facet_grid(phylum~Transition_history, scales = "free", space = "free",
             labeller = labeller(Transition_history = ge.lables.bar))+
  scale_y_continuous(labels = scientific) +
        labs(y = "Relative Abundnace (%)",x =" ", size = 12, fill = "Best classification") +
  theme_bw()+ theme(legend.position = "right", axis.text.x=element_blank(),
                    axis.ticks = element_blank(),
              panel.grid.major = element_blank(), panel.grid.minor = element_blank(),
```

```
              legend.key.size = unit(0.2, "cm"),legend.text = element_text(size = 10),
              legend.title = element_text(size = 12, face = "bold"),
              strip.background = element_blank(),
              strip.text.x = element_text(size= 12, face = "bold"),
              strip.text.y = element_text(size= 12, face = "bold",angle = 360),
              axis.title.y = element_text( size=13, face="bold"))


eg.lables.bar = c("Longterm_Exotic"="Exotic","Recent_E->G"= "Recent",
                  "Historic_E->G"="Historic","Longterm_Grassland"= "Grassland")
levels(sample_data(shared.EG)$Transition_history)
sample_data(shared.EG)$Transition_history <-
  factor(sample_data(shared.EG)$Transition_history,
         levels = c("Longterm_Exotic",  "Recent_E->G",
                    "Historic_E->G","Longterm_Grassland"))
clean.names.EG = c("Acidobacteriae ASV167", "Micrococcaceae ASV177",
                   "Subgroup 2 ASV890","Gaiella ASV911","Pir4 lineage ASV1327",
                   "Micrococcaceae ASV1509","Haliangium ASV1710","IMCC26256 ASV2480",
                   "Burkholderia ASV3877","Planctomycetes ASV4275")


plot_bar(shared.EG, fill="taxa_ASV") +
  facet_grid(phylum~Transition_history, scales = "free", space = "free",
             labeller = labeller(Transition_history = eg.lables.bar))+
  scale_y_continuous(labels = scientific) +
         scale_fill_brewer(palette = "Set3", labels=clean.names.EG) +
        labs(y = "Relative Abundnace (%)",x =" ",
             size = 12, fill = "Best classification") +
  theme_bw()+
        theme(legend.position = "right", axis.text.x=element_blank(),
              axis.ticks = element_blank(),
              panel.grid.major = element_blank(), panel.grid.minor = element_blank(),
              legend.key.size = unit(0.2, "cm"),legend.text = element_text(size = 10),
              legend.title = element_text(size = 12, face = "bold"),
              strip.background = element_blank(),
              strip.text.x = element_text(size= 12, face = "bold"),
              strip.text.y = element_text(size= 12, face = "bold",angle = 360),
              axis.title.y = element_text( size=13, face="bold"))
save_plot("ind.bar.16S.EG.png", last_plot(), base_width = 9 , base_height = 5)


gh.lables.bar = c("Longterm_Grassland"="Grassland","Recent_G->H"="Recent",
                  "Historic_G->H"="Historic",
                  "Longterm_Horticulture"= "Horticulture")
levels(sample_data(shared.GH)$Transition_history)
sample_data(shared.GH)$Transition_history <-
  factor(sample_data(shared.GH)$Transition_history,
         levels =c("Longterm_Grassland","Recent_G->H",
                   "Historic_G->H","Longterm_Horticulture"))
plot_bar(shared.GH, fill="taxa_ASV") +
  facet_grid(phylum~Transition_history, scales = "free",space = "free",
             labeller = labeller(Transition_history = gh.lables.bar))+
  scale_y_continuous(labels = scientific) +
```

```
        scale_fill_brewer(palette = "Dark2") +
        labs(y = "Relative Abundnace (%)",x =" ", size = 12, fill = "Best classification") +
  theme_bw()+
        theme(legend.position = "right", axis.text.x=element_blank(),
              axis.ticks = element_blank(),
              panel.grid.major = element_blank(), panel.grid.minor = element_blank(),
              legend.key.size = unit(0.2, "cm"),legend.text = element_text(size = 10),
              legend.title = element_text(size = 12, face = "bold"),
              strip.background = element_blank(),
              strip.text.x = element_text(size= 12, face = "bold"),
              strip.text.y = element_text(size= 12, face = "bold",angle = 360),
              axis.title.y = element_text( size=13, face="bold"))
```

Taxa related to aromatic compound, lysine and geraniol degradation

```
css.joined = subset_samples(data_norm_RA.all, Transition_spec == "Grassland" |
                              Transition_spec == "Exotic" |Transition_spec == "G->E" |
                              Transition_spec == "E->G")
ps.css.degraders = subset_taxa(css.joined, genus %in% degradaers)
otu_table(ps.css.degraders) = t(otu_table(ps.css.degraders))
#Mean abundances for each group
TGroup <- tax_glom(ps.css.degraders, taxrank = "genus")
SampleType = sample_data(TGroup)$Transition_spec
table(SampleType)
mean_PGroup = sapply(levels(SampleType),function(i){
  rowMeans(otu_table(TGroup)[,SampleType==i])
})
sd_PGroup = sapply(levels(SampleType),function(i){
  rowSde(otu_table(TGroup)[,SampleType==i])
})
phy = tax_table(TGroup)[rownames(mean_PGroup ),"genus"]
rownames(mean_PGroup) = phy
rownames(sd_PGroup) = phy
head(sort(rowMeans(mean_PGroup),decreasing=TRUE))
head(sort(rowMeans(sd_PGroup),decreasing=TRUE))
#Extract names of significantly different groups
deg.mean <- as.data.frame(mean_PGroup)
deg.st <- as.data.frame(sd_PGroup)
setDT(deg.mean, keep.rownames = "genus")
long.mean.t <- melt(setDT(deg.mean), id.vars = "genus", variable.name = "LandUse")
setDT(deg.st , keep.rownames = "genus")
long.sd.t <- melt(setDT(deg.st), id.vars = "genus", variable.name = "LandUse")
names(long.mean.t)[3] <- "mean"
names(long.sd.t)[3] <- "sd"
deg.mean.sd = as.data.frame(cbind(long.mean.t,long.sd.t))
deg.mean.sd = deg.mean.sd[,-1]

ggplot(subset(deg.mean.sd, LandUse == "Exotic" | LandUse == "Grassland"),
       aes(x = genus, y = mean, fill = LandUse)) +
  geom_bar(stat="identity", position=position_dodge()) + theme_bw() +
        geom_errorbar(aes(ymin=mean-sd, ymax=mean+sd),
                      width=.2,position=position_dodge(.9))+
    scale_fill_manual(values = c("orangered1","mediumseagreen"),
```

```
                      labels=c('Exotic forest', 'Grassland'))+
  labs(y="Relative abundance %" , x = "")+
    theme(panel.grid.major = element_blank(), panel.grid.minor = element_blank(),
       axis.text.x = element_text(angle = 30, hjust=1, size = 15, colour = "black"),
      panel.border = element_rect(linetype = "solid", colour = "black", size=0.6),
       axis.title = element_text(size= 15),
      axis.text.y = element_text(size = 15, colour = "black"),
      strip.background = element_blank(), strip.text = element_text(size=16, face ="bold"),
      legend.title = element_blank(),legend.text = element_text(size = 16),
      legend.position="top", legend.box = "horizontal", legend.justification='left')
```

Variance partitioning

```
library(vegan)
library(labdsv)
library(ggplot2)
library(tidyverse)
library(Manu)
library(phyloseq)
###Load all data
asv<-as.data.frame(as(otu_table(data_norm_RA.all), "matrix"))
asv<-asv[,colSums(asv[])>0]
meta = data.frame(sample_data(data_norm_RA.all))
site <- meta[,5:8]
chem<-meta[,15:24]
climate<-meta[,10:14]
xy<-meta[,5:6]
### CLIMATE
climate.stand<- decostand(climate,MARGIN=2,method="standardize")
### SOILS
chem.stand <- decostand(chem,MARGIN=2,method="standardize")
###Format bacterial data
## ASV - DM
seq.dm <- as.matrix(vegdist(asv,method="bray"))
pco.seq.dm <- pco(seq.dm)
plot(pco.seq.dm)
## ASV - PA
seq.pam <- as.data.frame(ifelse(asv>0,1,0))
seq.rich <- rowSums(seq.pam)
hist(seq.rich, main="Number of ASVs per sample")
### Spatial MEMs
### SPACE: TREND SURFACE
space <- xy
names(space)<-c("x", "y")
space$x2 <- space$x*space$x
space$y2 <- space$y*space$y
space$xy <- space$x*space$y
space$x2y <- space$x*space$x*space$y
space$xy2 <- space$x*space$y*space$y
#space1 <- space
space1 <- space[,c("x","y","x2","y2","xy")]
space <- decostand(space,MARGIN=2,method="standardize")
names(space) <- c("x","y","x2","y2","xy","x2y","xy2")
```

```r
### SPACE: MEMs
## calculate the MEMs for the sites
geo_dist <- vegdist(xy,method="euclidean") # Geographic distances
geo.PCNM <- pcnm(geo_dist,dist.ret=FALSE)
geo.pcnm <- as.data.frame(geo.PCNM$vectors)
dim(geo.pcnm)
## Select significant MEMS for COMMUNITY DATASETS
## Select significant MEMS for RICHNESS DATASETS
mod0.geo.pcnm <- capscale(seq.rich~1,data=geo.pcnm)
mod1.geo.pcnm <- capscale(seq.rich~.,data=geo.pcnm)
mod.step.geo.pcnm <- ordistep(mod0.geo.pcnm,scope=formula(mod1.geo.pcnm),
                              direction = "forward",perm.max=200,Pin=0.05)
seq.rich.varselect.pcnm.list <- names(mod.step.geo.pcnm[[6]]$envcentre) #selected MEMs
space_seq.rich <- as.matrix(geo.pcnm[seq.rich.varselect.pcnm.list])
## Select significant MEMS for DISSIMILARITY MATRIX
mod0.geo.pcnm <- capscale(seq.dm~1,data=geo.pcnm)
mod1.geo.pcnm <- capscale(seq.dm~.,data=geo.pcnm)
mod.step.geo.pcnm <- ordistep(mod0.geo.pcnm,scope=formula(mod1.geo.pcnm),
                              direction = "forward",perm.max=200,Pin=0.05)
seq.dm.varselect.pcnm.list <- names(mod.step.geo.pcnm[[6]]$envcentre) #selected MEMs
space_seq.dm <- as.matrix(geo.pcnm[seq.dm.varselect.pcnm.list])

vp.dm<- varpart4.MEM(seq.dm,site,chem.stand, climate.stand,space_seq.dm)
vp.rich <- varpart4.MEM(seq.rich,site,chem.stand,climate.stand,space_seq.rich)
```
